# Assessment of the association between genetic factors regulating thyroid function and microvascular complications in diabetes: A two-sample Mendelian randomization study in the European population

**DOI:** 10.3389/fendo.2023.1126339

**Published:** 2023-02-28

**Authors:** Hongdian Li, Mingxuan Li, Shaoning Dong, Sai Zhang, Ao Dong, Mianzhi Zhang

**Affiliations:** ^1^ Dongfang Hospital, Beijing University of Chinese Medicine, Beijing, China; ^2^ Beijing Hospital of Traditional Chinese Medicine, Capital Medical University, Beijing, China; ^3^ Department of Nephrology, Tianjin academy of Traditional Chinese Medicine Affiliated Hospital, Tianjin, China

**Keywords:** thyroid function, diabetic kidney disease, diabetic retinopathy, Mendelian randomization, kidney function

## Abstract

**Background:**

Observational studies have identified a possible link between thyroid function and diabetic microangiopathy, specifically in diabetic kidney disease (DKD) and diabetic retinopathy (DR). However, it is unclear whether this association reflects a causal relationship.

**Objective:**

To assess the potential direct effect of thyroid characteristics on DKD and DR based on Mendelian randomization (MR).

**Methods:**

We conducted an MR study using genetic variants as an instrument associated with thyroid function to examine the causal effects on DKD and DR. The study included the analysis of 4 exposure factors associated with thyroid hormone regulation and 5 outcomes. Genomewide significant variants were used as instruments for standardized freethyroxine (FT4) and thyroid-stimulating hormone (TSH) levels within the reference range, standardized free triiodothyronine (FT3):FT4 ratio, and standardized thyroid peroxidase antibody (TPOAB) levels. The primary outcomes were DKD and DR events, and secondary outcomes were estimated glomerular filtration rate (eGFR), urinary albumin-to-creatinine ratio (ACR) in diabetes, and proliferative diabetic retinopathy (PDR). Satisfying the 3 MR core assumptions, the inverse-variance weighted technique was used as the primary analysis, and sensitivity analysis was performed using MR-Egger, weighted median, and MR pleiotropy residual sum and outlier techniques.

**Results:**

All outcome and exposure instruments were selected from publicly available GWAS data conducted in European populations. In inverse-variance weighted random-effects MR, gene-based TSH with in the reference range was associated with DKD (OR 1.44; 95%CI 1.04, 2.41; P = 0.033) and eGFR (β: -0.031; 95%CI: -0.063, -0.001; P = 0.047). Gene-based increased FT3:FT4 ratio, decreased FT4 with in the reference range were associated with increased ACR with inverse-variance weighted random-effects β of 0.178 (95%CI: 0.004, 0.353; P = 0.046) and -0.078 (95%CI: -0.142, -0.014; P = 0.017), respectively, and robust to tests of horizontal pleiotropy. However, all thyroid hormone instruments were not associated with DR and PDR at the genetic level.

**Conclusion:**

In diabetic patients, an elevated TSH within the reference range was linked to a greater risk of DKD and decreased eGFR. Similarly, decreased FT4 and an increased FT3:FT4 ratio within the reference range were associated with increased ACR in diabetic patients. However, gene-based thyroid hormones were not associated with DR, indicating a possible pathway involving the thyroid-islet-renal axis. However, larger population studies are needed to further validate this conclusion.

## Introduction

Diabetic Kidney Disease (DKD) and Diabetic Retinopathy (DR) are among the most crucial microvascular lesions in diabetes, and frequently occur concomitantly (1). The growing global prevalence of diabetes is also leading to an increase in the population affected by DKD and DR. DKD is a major contributor to End-stage Renal Disease (ESRD), with an estimated 91% of all new cases of diabetes-related ESRD attributed to Type 2 Diabetes Mellitus (T2DM) according to the US Renal Data System (2). In developed countries, nearly 40% of DKD patients eventually require dialysis (3). DR is a leading cause of blindness among adults, and it is projected that over 200 million people worldwide will develop DR by 2040 (4). The complex chain reaction initiated by diabetes may be attributed to the buildup of Advanced Glycation End Products (AGEs) and increased erythrocyte adhesion to endothelial cells, which is a key pathogenic mechanism of the vascular complications associated with T2DM (5, 6). There may also be potential associations with other factors, including thyroid function.

Thyroid-related diseases and diabetes mellitus are two of the most significant metabolic disorders that have a well-documented association (7–9). The presence of thyroid hormone receptors in the vascular endothelial tissue means that alterations in circulating thyroid hormone levels can contribute to the development and progression of vascular disease (10). Hence, the influence of thyroid function on diabetic microvascular complications is attracting increased attention. The regulation of thyroid function is intricate and involves multiple components, including the pituitary and hypothalamus, feedback mechanisms, and the thyroid’s own characteristics and functions. The pituitary gland produces and releases thyroid-stimulating hormone (TSH), which stimulates the release of thyroxine from the thyroid gland. Thyroxine circulates in the body in a balanced state between its isolated protein-bound form and the bioavailable free form, referred to as free thyroxine (FT4). In both the thyroid and peripheral tissues, FT4 is converted to the active form of triiodothyronine (FT3), which can be assessed by the FT3:FT4 ratio in the circulation (11). Thyroid peroxidase antibodies (TPOAB), a biomarker of autoimmune thyroid disease, is a sensitive indicator of thyroid function and diverse physiological responses (12). Observational cross-sectional studies have revealed an independent association between the presence of subclinical hypothyroidism (SCH) and DKD (13). The results showed that DKD was negatively correlated with levels of free triiodothyronine (FT3) and free thyroxine (FT4), and positively correlated with thyroid-stimulating hormone (TSH) levels. In addition, low to normal levels of thyroid hormones were associated with the presence of massive albuminuria, and TSH and FT3 were found to be potential predictors of DKD (14–16). The hypothalamic-pituitary-thyroid axis plays a critical role in retinal development and increases retinal vascular density (17, 18). A recent study conducted in China explored the relationship between FT3 levels and DR in patients with T2DM who have normal thyroid function. Results showed a negative association between FT3 levels and DR in these patients (19). This observation was further supported by the finding that treatment of T2DM patients with thyroid hormones was associated with improvement in retinopathy (20). DKD and DR are interdependent risk factors for one another, with evidence suggesting a shared relationship with thyroid function (21–23). However, despite being influenced by common factors, current population-based clinical studies lack sufficient evidence to establish a direct causal link between thyroid function and the development of DKD and DR. Further research is needed to fully understand the underlying mechanisms and establish a clear relationship between these conditions.

Mendelian randomization (MR) is an analytical approach that leverages genetic variation to assess the causal relationship between independent and outcome variables in observational studies. This method is considered to be less susceptible to confounding or reverse causality compared to traditional observational analyses (24). The premise of MR is that if 7thyroid hormone levels have a direct impact on the development of DKD and DR, then genetic variants affecting thyroid function should also be associated with DKD and DR, with the magnitude of this association being consistent with the observed relationships.

The aim of this study was to investigate the potential causal relationship between thyroid function and DKD and DR using two-sample MR analysis. To complement the findings of MR, we conducted a further analysis using estimated glomerular filtration rate (eGFR) and urinary albumin to creatinine ratio (ACR) in patients with DM as secondary outcomes. The purpose of this complementary analysis was to provide additional evidence on the causal effect of thyroid function on DKD. Additionally, our study aimed to explore the causal effect of thyroid hormones on proliferative diabetic retinopathy (PDR), with the intention of identifying the need for increased protection of thyroid function in patients with severe DR lesions.

## Methods

### Data sources

In a genome-wide association study (GWAS) of thyroid traits among individuals of European ancestry, instrument-exposure associations were identified for FT4, FT3:FT4 ratio, TSH, and TPOAB based on single nucleotide polymorphisms (SNPs). Only SNPs that reached genome-wide significance levels (p < 5 × 10^-8^) were considered in this European population. To avoid linkage disequilibrium reactions and the potential double counting of similar genes within a certain range, screening conditions were set at R^2^ < 0.001 and kb = 10,000. A GWAS of reference range FT4 levels was conducted using data from 72,167 European subjects, as published by Teumer et al. The study identified 31 genetic loci with significant associations with reference range FT4 levels, implicating a role for these loci in thyroid development, physiological function and transport of thyroid hormones, as well as metabolism of these hormones (25). In a study conducted by Panicker et al., GWAS data for the FT3:FT4 ratio was obtained. The results revealed that a SNP, rs2235544, located in the DIO1 gene was significantly associated with the FT3:FT4 ratio at a genome-wide level. The researchers found that the presence of the C allele at this SNP locus was associated with an increase in the activity of deiodinase 1, leading to an elevated FT3:FT4 ratio. This elevated ratio was found to affect the physiological function of the thyroid gland (26). Genetic susceptibility loci associated with TSH levels in the reference range were derived from two studies that identified a total of 61 susceptibility loci associated with TSH levels in the reference range, and these loci were strongly associated with the development of thyroid cancer and goitre (27, 28). Medici et al. and Schultheiss et al. conducted two GWAS studies that identified a total of five susceptibility loci associated with TPOAB that predicted which TPOAB positivity predisposed to the development of clinical thyroid dysfunction (29, 30). The studies were granted ethical clearance by the institutional review board. **Table 1** displays the characteristics of the results from the Genome-Wide Association Study (GWAS).

**Table 1:** The characteristics of GWAS studies on the outcomes.

Outcomes	Consortium	Sample size	Ethnicity	Web source
DKD	Finngen	3,676 cases and 283,456 controls	European	https://www.finngen.fi/en/access_results
DR	Finngen	8,942 cases and 283,545 controls	European	https://www.finngen.fi/en/access_results
PDR	Finngen	8,383 cases and 329,756 controls	European	https://www.finngen.fi/en/access_results
eGFR in diabetes	–	55,114 individuals	European	PMID: 26831199
ACR in diabetes	CKDgen	5,825 cases and 46061 controls	European	http://ckdgen.imbi.uni-freiburg.de/ PMID: 26631737

DKD, diabetic kidney disease; DR, diabetic retinopathy; PDR, proliferative diabetic retinopathy; eGFR, estimated glomerular filtration rate; ACR, urinary albumin-to-creatinine ratio.

The outcome measures were drawn from publicly available genetic association studies conducted in European populations. The primary outcomes were DKD and DR, and the raw data was obtained from the Finngen database (r8) (31), which included patients with all types of diabetes. The genetic association study cohort for DKD consisted of 3,676 cases and 283,456 controls, while the cohort for DR consisted of 8,942 cases and 283,545 controls. Secondary outcomes included eGFR and ACR in individuals with diabetes and PDR. The genetic association study data for eGFR in individuals with diabetes was obtained from the study by Pattaro et al. published in 2016, which consisted of 39 studies and a total of 55,114 individuals with diabetes. eGFR was calculated using the four-variable Modification of Diet in Renal Disease Study Equation (32). The genetic association study data for ACR in 5,825 individuals with diabetes and 46061 controls was obtained from the study by Teumer et al. published in 2016, and ACR was calculated as the ratio of urinary albumin to urinary creatinine to account for variations in urine concentration (33). The genetic association study cohort for PDR was also obtained from the Finngen database, consisting of 8,383 cases and 329,756 controls (31).

### Mendelian randomization analysis

**Figure 1:**
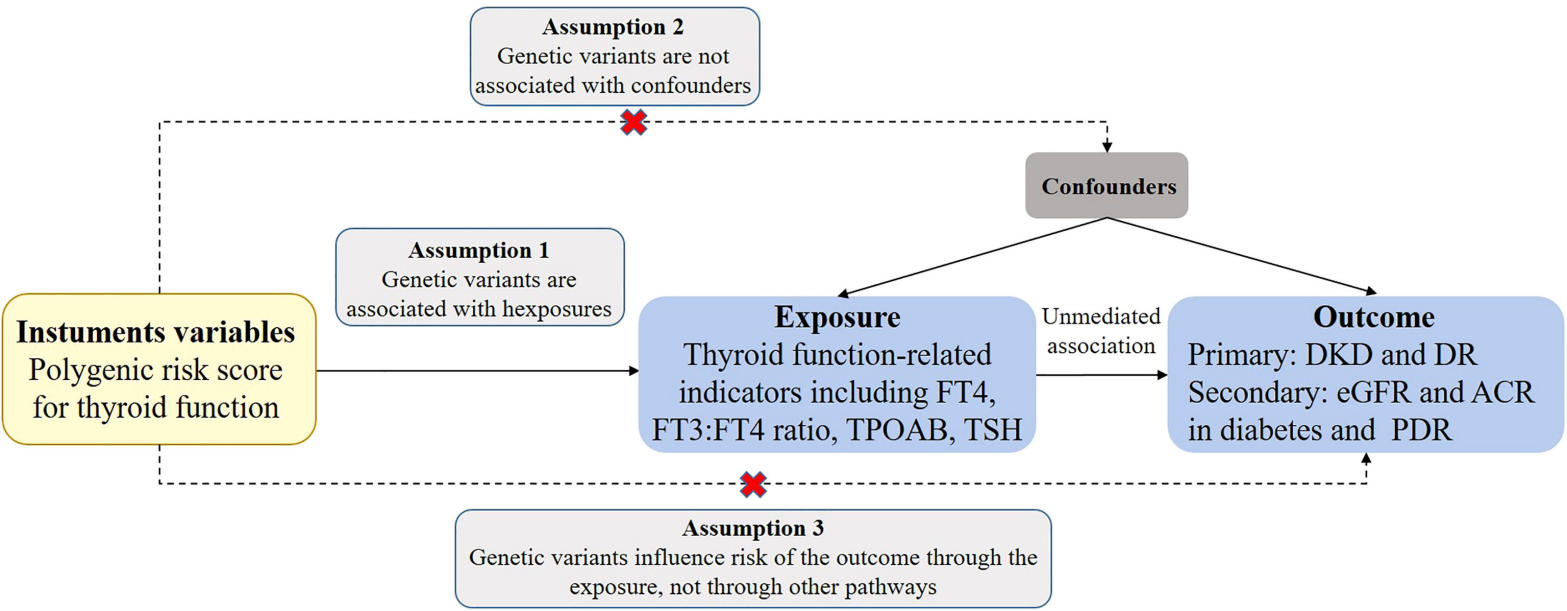
Assumptions of a Mendelian randomization analysis for thyroid function and risk of DKD and DR. Broken lines represent potential pleiotropic or direct causal effects between variables that would violate Mendelian randomization assumptions. FT4, free thyroxine; FT3, free triiodothyronine; TSH, thyroid-stimulating hormone; TPOAB, thyroid peroxidase antibodies; DKD, diabetic kidney disease; DR, diabetic retinopathy; PDR, proliferative diabetic retinopathy; eGFR, estimated glomerular filtration rate; ACR, urinary albumin-to-creatinine ratio.

In this study, the MR analysis tool was utilized to determine the causal relationship between thyroid function and various outcome indicators. SNPs were used as instrumental variables to estimate the causal effect. To ensure the validity of the results, three core assumptions were made: first, the genetic variations were associated with exposure factors; second, the genetic variations were independent of confounding factors; and third, the genetic variations only had an effect on the outcome through the exposure and not through any other pathways (**Figure 1**). To obtain the primary overall instrumental estimate, an Inverse-Variance Weighted Fixed-Effects MR method was employed, which considered all genetic variants as valid instruments without any pleiotropy. The individual instrumental estimates and their standard errors were then combined using the Inverse-Variance Weighted (IVW) method to produce the final MR results (34). In order to mitigate the impact of horizontal pleiotropy, where genetic variation has a significant effect on results *via* pathways other than exposure, the current study utilized three statistical methods: IVW random-effects (IVW-RE), weighted median (WM), and MR-Egger methods (35, 36). To ensure robust results, a range of sensitivity analyses were conducted, including heterogeneity tests, horizontal pleiotropy tests, funnel plot analysis, and a leave-1-variant-out analysis using the IVW-RE method, where one variable was excluded from the analysis in each iteration. The individual instrumental variables were analyzed using the Instrumental Variable Ratio (Wald) estimator.

In this study, the strength of all instruments was evaluated using the F statistic (calculated as F = β^2^_exposure_/SE^2^_exposure_). This approach was taken to ensure that weak instrumental variables do not influence the results of the causal estimation. The results showed that there were no weak instrumental variables in the study, as the F-statistic ranges for FT4, TSH, TPOAB and FT3:FT4ratio tools were 29-394, 29-576, 10-19, and 21, respectively. To check for the presence of pleiotropy, several sensitivity analyses were performed. The Q statistics (Cochran’s Q for IVW and Rücker’s Q for MR-Egger) were calculated to assess heterogeneity in individual causal effects, with p-values less than 0.05 indicating the presence of heterogeneity (37). The MR-Egger intercept term was used to evaluate horizontal pleiotropy, and a deviation from zero indicates directional pleiotropy. The slope of the MR-Egger regression was used to provide a valid MR estimate in the presence of horizontal multiplicity (38, 39). A complementary weighted median method was also employed, which assumes that at least 50% of the inverse-variance is valid and ranks the inverse of the weighted variance of MR estimates for each inverse-variance (34). The MR pleiotropy residual sum and outlier (MR-PRESSO) outlier test was performed to correct for horizontal pleiotropy *via* outlier removal (40). The effect values are expressed as β when the ending variable is a continuous variable and as odds ratio (OR) when it is a dichotomous variable.

## Results

### MR estimates of causal effects of thyroid function on DKD

**Figure 2:**
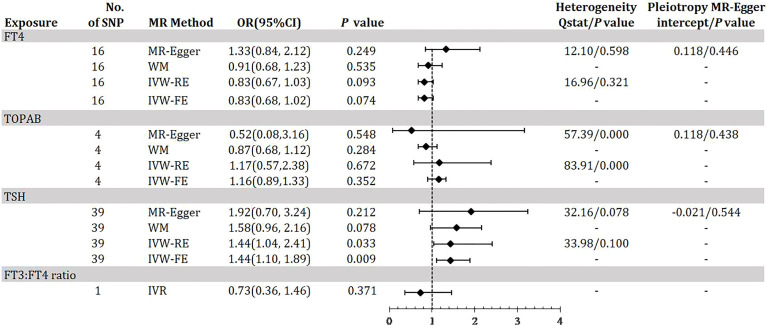
Odds ratio for association of genetically predicted thyroid function with DKD. FT4, free thyroxine; FT4, free thyroxine; FT3, free triiodothyronine; TSH, thyroid-stimulating hormone; TPOAB, thyroid peroxidase antibodies; DKD, diabetic kidney disease; CI, confidence internal; OR, odds ratio; IVW-FE, inverse-variance weighted fixed-effects MR; IVW-RE, inverse-variance weighted random-effects MR; MR, mendelian randomization; WM, weighted median; IVR, instrumental variable ratio (Wald) estimator; SNP, single-nucleotide polymorphism. *P* value for heterogeneity based on Cochran’s Q statistic for IVW, and Rücker’s Q for MR-Egger.

**Figure 2** demonstrates the MR estimation between thyroid function and DKD. After conditional screening and MR-PRESSO test, we identified 16 of 31 SNPs for FT4 within the reference range, 1 of 1 SNP for FT3:FT4 ratio (the DIO1, rs2235544), 39 of 61SNPs for TSH within the reference range, 4 of 5 SNPs for TPOAB concentration. Genetically predicted TSH was associated with DKD with an IVW-RE OR of 1.44 (95% CI 1.04-2.41; P = 0.033) (**Figure 2**, **eTable 2**, **eFigure 2A, B** in the **Supplement**) with similar and a more significant result in IVW-FE (OR=1.44, 95% CI, 1.10-1.89; P = 0.009), and the results were similar with the MR-Egger and WM analyses. We did not find a significant risk association between FT4 (OR=0.83, 95% CI 0.67-1.03; P = 0.093) and TPOAB (OR=1.17, 95% CI 0.57-2.38; P = 0.672) in the reference range and DKD (**Figure 2**, **eTable 1, 3**, **eFigure 1A, B**, **3A, B** in the **Supplement**). A genetically predicted 1 SD–increase in FT3:FT4 ratio by the C allele was not associated with increased DKD with an OR of 0.73 (95% CI 0.36-1.46; P =0.371) (**Figure 2**, **eTable 1** in the **Supplement**). There was some evidence of heterogeneity based on Q-statistic (Q-value _IVW_ = 83.91, P-value = 0.000; Q-value _MR-Egger_ = 57.39, P-value = 0.00) for the TPOAB analysis. Consequently, weights were penalized for the IVW method. The FT4 and TSH variants were distributed symmetrically about the combined effect size in the funnel plot (**eFigure 1C**, **2C** in the **Supplement**), and MR-Egger did not show evidence of horizontal pleiotropy (FT4: P _for MR-Egger_ = 0.446; TSH: P _for MR-Egger_ = 0.544) (**Figure 2**). Because TPOAB has fewer relevant instrumental variables, the causal effects of its funnel plot are not symmetric (**eFigure 3C** in the **Supplement**). However, the MR-Egger intercept that we performed did not show evidence of horizontal pleiotropy (P _for MR-Egger_ = 0.438) (**Figure 2**). These results show that no directional pleiotropic effects are present in our study. The leave-one-out test did not identify any thyroid function-related variants that had a strong effect on the overall results (**eFigure 1D**,**2D**, **3** in the **Supplement**).

### MR estimates of causal effects of thyroid function on DR

**Figure 3:**
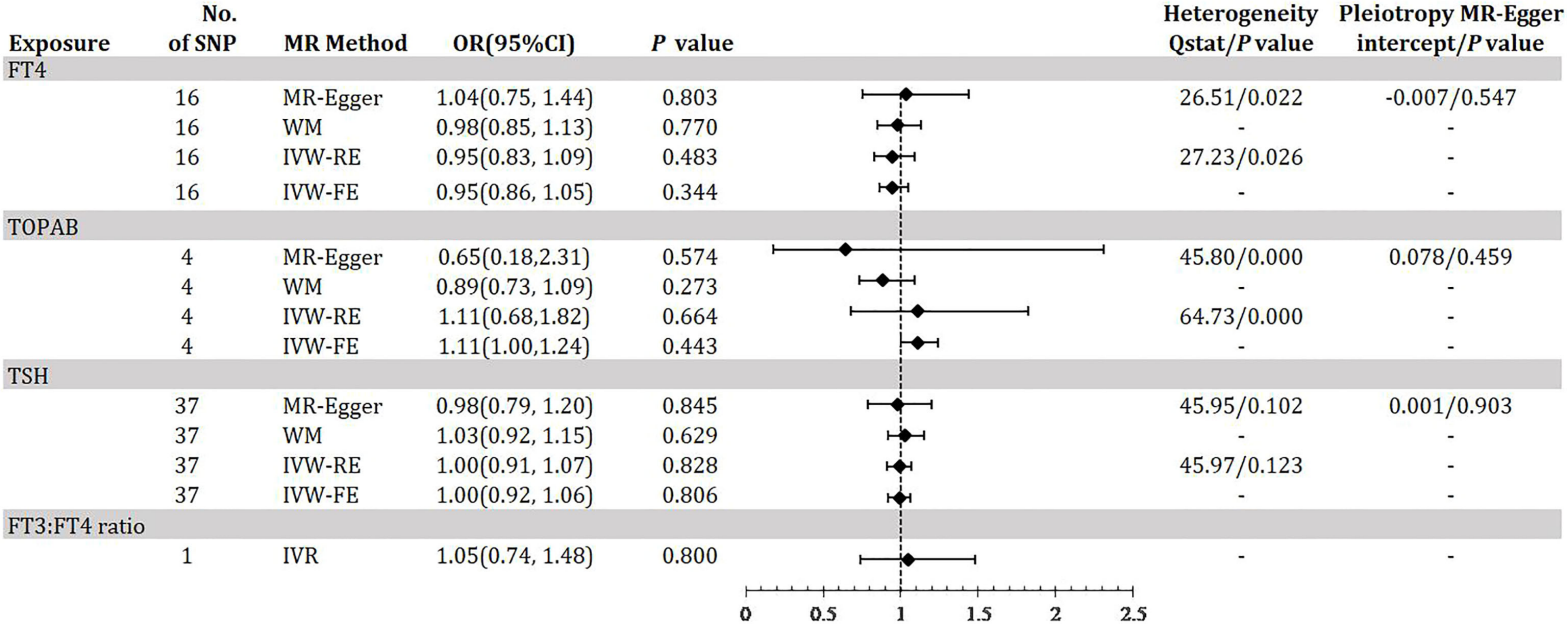
Odds ratio for association of genetically predicted thyroid function with DR. FT4, free thyroxine; FT4, free thyroxine; FT3, free triiodothyronine; TSH, thyroid-stimulating hormone; TPOAB, thyroid peroxidase antibodies; DR, diabetic retinopathy; CI, confidence internal; OR, odds ratio; IVW-FE, inverse-variance weighted fixed-effects MR; IVW-RE, inverse-variance weighted random-effects MR; MR, mendelian randomization; WM, weighted median; IVR, instrumental variable ratio (Wald) estimator; SNP, single-nucleotide polymorphism. *P* value for heterogeneity based on Cochran’s Q statistic for IVW, and Rücker’s Q for MR-Egger.

**Figure 3** demonstrates the MR estimation between thyroid function and DR. After conditional screening and MR-PRESSO test, we identified 16 of 31 SNPs for FT4within the reference range, 1of1SNP forFT3:FT4 ratio (the DIO1, rs2235544), 37 of 61SNPs for TSH within the reference range, 4 of 5 SNPs for TPOAB concentration. We did not find any statistically significant genetic risk association between thyroid function-related instruments and DR by IVW-RE (FT4: OR=0.95, 95% CI 0.83-1.09; P=0.483; TPOAB: OR=1.11, 95% CI 0.68-1.82; P=0.664; TSH: OR=1.00, 95% CI 0.91-1.07; P=0.828; **Figure 3**, **eTable 4-6**, **eFigure 4-6, A, B** in the **Supplement**), which is similar to the results of IVW-FE, MR-Egger and WM analyses. A genetically predicted 1 SD–decrease in FT3:FT4 ratio by the C allele was not associated with increased DR with an OR of 1.05 (95% CI, 0.74-1.48; P =0.800) (**Figure 2**, **eTable 4** in the **Supplement**). There was some evidence of heterogeneity in the analysis regarding FT4 (Q-value _IVW_ = 27.23, P-value = 0.026; Q-value _MR-Egger_ = 26.51, P-value = 0.022) and TPOAB (Q-value _IVW_ = 64.73, P-value = 0.000; Q-value _MR-Egger_ = 45.80, P-value = 0.000) according to Q-statistics. The FT4 and TSH variants were distributed symmetrically about the combined effect size in the funnel plot (**eFigure 4C, e5C** in the **Supplement**), and MR-Egger did not show evidence of horizontal pleiotropy (FT4: P _for MR-Egger_ = 0.547; TSH: P _for MR-Egger_ = 0.459) (**Figure 3**). Because TPOAB has fewer relevant instrumental variables, the causal effects of its funnel plot are not symmetric (**eFigure 6C** in the **Supplement**). However, the MR-Egger intercept that we performed did not show evidence of horizontal pleiotropy (P _for MR-Egger_ = 0.903) (**Figure 3**). These results show that no directional pleiotropic effects are present in DR study. The leave-one-out test did not identify any thyroid function-related variants that had a strong effect on the overall results (**eFigure 4D**, **5D**, **6D** in the **Supplement**).

### MR estimates of causal effects of thyroid function on eGFR and ACR in diabetes

**Figure 4:**
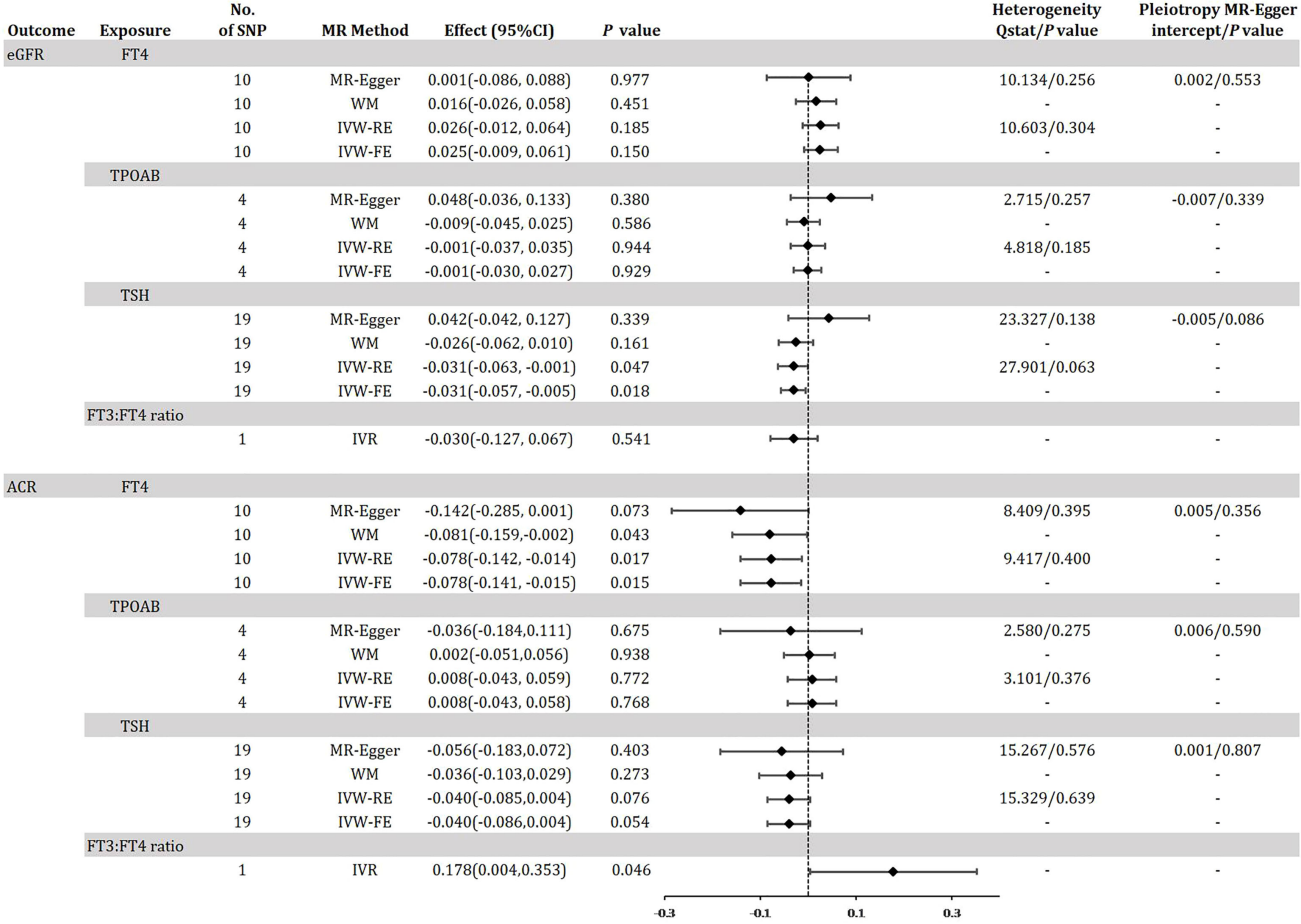
Odds ratio for association of genetically predicted thyroid function with eGFR and ACR in diabetes. FT4, free thyroxine; FT4, free thyroxine; FT3, free triiodothyronine; TSH, thyroid-stimulating hormone; TPOAB, thyroid peroxidase antibodies; eGFR, estimated glomerular filtration rate; ACR, urinary albumin-to-creatinine ratio; CI, confidence internal; OR, odds ratio; IVW-FE, inverse-variance weighted fixed-effects MR; IVW-RE, inverse-variance weighted random-effects MR; MR, mendelian randomization; WM, weighted median; IVR, instrumental variable ratio (Wald) estimator; SNP, single-nucleotide polymorphism. *P* value for heterogeneity based on Cochran’s Q statistic for IVW, and Rücker’s Q for MR-Egger.

**Figure 4** shows the MR estimation between thyroid function and renal impairment indicators eGFR and ACR in diabetic patients to further reflect the genetic association with DKD. After linkage disequilibrium screening and MR-PRESSO assay, we identified a total of 10 out of 31 SNPs for FT4 within the reference range, 1 SNP for FT3:FT4 ratio (DIO1, rs2235544), 19 out of 61 SNPs for TSH in the reference range, and 4 SNPs for TPOAB concentration. We found that IVW-RE genetically predicted TSH was negatively correlated with eGFR, i.e., for 1-sd increase in TSH in the reference range, eGFR decreased by 0.031 (95% CI -0.063, -0.001; P=0.047), a result that was more significant in IVW-FE (Effect: -0.031, 95% CI -0.057, - 0.005; P=0.018) was more significant and similar to the results of WM analysis (**Figure 4**, **eTable 8**, **eFigure 8A, B** in the **Supplement**). No significant association between other thyroid function predictors and eGFR was found (**eTable 7**,**9**, **7 A-B**, **9 A-B** in the **Supplement**). The IVW-RE OR for ACR per SD of FT4 within the reference range was -0.078 (95% CI -0.142, -0.014; P = 0.017) (**Figure 4**, **eFigure 10 A-B** in the **Supplement**). Results were similar for IVW-FE (P = 0.015), MR-Egger (P = 0.073) and WM (P = 0.043) analysis. Notably, we found that a 1 SD increase in the FT3:FT4 ratio of the C allele was associated with an increase in ACR with an effect of 0.178 (95% CI, 0.004-0.353; P=0.046), a result consistent with the trend in FT4 results (**eTable 10** in the Supplement). There was no evidence of heterogeneity based on Q-statistic for analyses of all thyroid function indicators (P _for het >_0.05). In our study, we found no evidence of pleiotropy, i.e., the *P* values for the pleiotropy of FT4, TSH and TPOAB were all greater than 0.05(**eFigure 7-12C** in the **Supplement**). The leave-one-out test did not identify any thyroid function-related variants that had a strong effect on the overall results (**eFigure 7-12D** in the **Supplement**).

### MR estimates of causal effects of thyroid function on PDR

**Figure 5:**
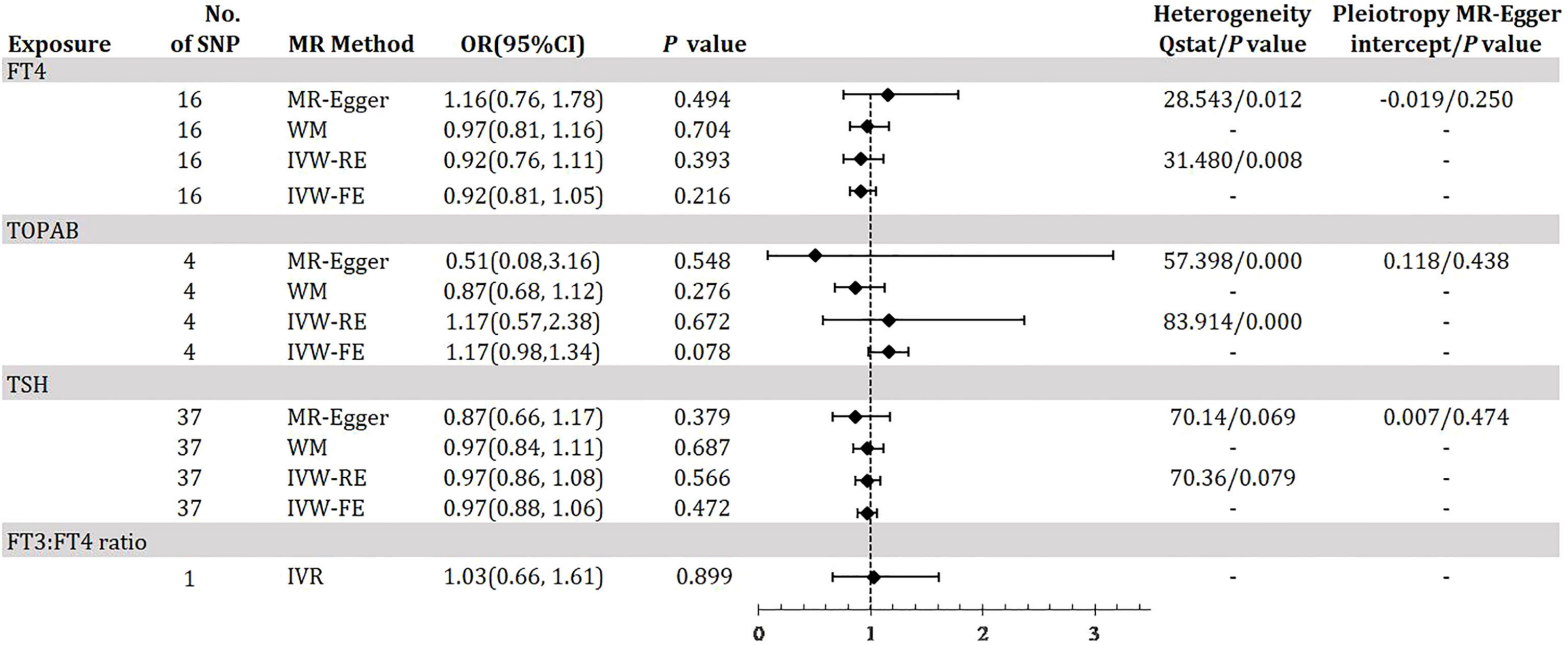
Odds ratio for association of genetically predicted thyroid function with PDR. FT4, free thyroxine; FT4, free thyroxine; FT3, free triiodothyronine; TSH, thyroid-stimulating hormone; TPOAB, thyroid peroxidase antibodies; PDR, proliferative diabetic retinopathy; CI, confidence internal; OR, odds ratio; IVW-FE, inverse-variance weighted fixed-effects MR; IVW-RE, inverse-variance weighted random-effects MR; MR, mendelian randomization; WM, weighted median; IVR, instrumental variable ratio (Wald) estimator; SNP, single-nucleotide polymorphism. *P* value for heterogeneity based on Cochran’s Q statistic for IVW, and Rücker’s Q for MR-Egger.

**Figure 5** demonstrates the MR estimation between thyroid function and PDR. We did not find any statistically significant genetic risk association between thyroid function-related instruments and DR (**eTable13-15**, **eFigure 13-15A, B** in the **Supplement**). The MR-Egger intercept that we performed did not show evidence of horizontal pleiotropy (C of **eFigure 13-15** in the Supplement), and the leave-one-out test did not identify any thyroid function-related variants that had a strong effect on the overall results (D of **eFigure 13-15** in the Supplement). These results are similar to the MR analysis of DR.

## Discussion

The current study, to the best of our knowledge, is the first to assess the relationship between thyroid function and diabetic microvascular complications, including DKD and DR, using MR analysis. Using publicly available GWAS data from European populations, we have made a novel finding that there is a potential genetic influence on TSH levels in the normal range that is associated with DKD, a hypothesis that was supported by the evidence in the eGFR. Furthermore, we examined the risk association between genetically determined thyroid function and ACR, and found that both FT4 and FT3:FT4 ratio may be genetic factors that are implicated in ACR. However, the combined genetic effect of all thyroid function indices did not provide strong evidence for a direct association with DR and PDR.

The thyroid-islet-renal axis represents a range of phenotypes that are dependent on phenotypes upstream of the axis and also on negative feedback mechanisms downstream. Both insulin and thyroid hormones are affected by autoimmune pathology, are part of the metabolic syndrome, and affect cellular metabolism. The pathophysiological association between diabetes and thyroid dysfunction is thought to be the result of the interaction of various biochemical, genetic and hormonal dysfunctions (41). As the most important microvascular complication of diabetes, the association between DKD and thyroid function is increasingly being demonstrated. We derived the presence of a genetic-based effect of thyroxine on DKD through multiple MR stratification analyses, based on unique and shared genetic tools, which is qualitatively different from extant epidemiological studies. Our study found that TSH levels in the reference range were positively associated with DKD risk and negatively associated with eGFR, i.e., elevated TSH may increase the risk of developing DKD as well as decrease eGFR. This result was confirmed in several observational studies. Renal function is directly correlated with thyroid function, as reflected by the positive correlation between TSH and serum creatinine and the negative correlation with eGFR (42, 43). Even when thyroid function is within the normal range, patients with DKD have higher TSH levels than diabetic patients without DKD (44, 45). A recent study reconfirmed that levothyroxine treatment reduced urinary albumin excretion in patients with early DKD with mildly elevated TSH levels and positive serum TPOAB (46) In addition, our study identified a risk association between FT3/FT4 and FT4 and ACR, and although this result was not statistically significant in DKD events and eGFR, the effect values showed a reliable and consistent trend. FT4 SNPs were derived from associations between individuals with FT4 levels in the reference range and no evidence of thyroid disease. In contrast, TSH instrumentation within the reference range is associated with hypothyroidism and hyperthyroidism. These differences may emphasize that normal variation in TSH and thyroid function drives the association of instrumentation with DKD. Another possible explanation is that although ACR and eGFR are the most commonly used clinical tools to assess chronic kidney disease, one study found no correlation between serum creatinine and ACR in patients with T2DM (47), so the association between thyroid function and them is informative but not determinative for the risk of DKD events. Clinical evidence found reduced FT4 and elevated TSH in the DKD population compared to non-DKD patients, and hypothyroidism was associated with increased ACR or reduced eGFR in patients with T2DM, and hypothyroid patients with T2DM exhibited higher ACR and urinary transferrin excretion (48), which is consistent with our results. The relationship between FT3:FT4 and DKD is unclear, but studies are currently being conducted in other areas related to diabetes. For example, a recent report from southern China found that low FT3/FT4 was associated with a poor prognosis of acute myocardial infarction in T2DM patients with normal thyroid function (49). Another MR study showed that genetically based high FT3/FT4 was associated with an increased risk of atrial fibrillation (12). In addition, a Belgian report showed that FT3:FT4 in late pregnancy was positively associated with gestational diabetes, adverse pregnancy outcomes and poor metabolic profile in the early postpartum period (7).

In our present study no correlation between thyroid hormones and DR was found, despite our stratification of DR. There is conflicting evidence regarding the association between DR and thyroid function (50–52), with some studies suggesting no significant association while others indicating a possible link. A decrease in thyroid hormone or SCH may increase the probability of DR, PDR, and diabetic macular edema (53–55). Lin et al. first retrospectively found that high TSH serum levels were associated with an increased prevalence of DR in diabetic patients, and then found *in vitro* that high glucose stimulated apoptosis and mitochondrial dysfunction in human peripapillary cells, which could be attributed to co-stimulation of glucose and high TSH (56). These studies supporting the association of thyroid function with DR are based on Asian populations, whereas our study was conducted in a European population. Clinical observational studies are difficult to control for confounders and multiple biases making it difficult to derive a direct causal association between exposure and outcome, therefore the relationship between thyroid function and DR needs to be further demonstrated in larger well-designed trials.

The pathogenesis of abnormal thyroid function is associated with endothelial dysfunction, hyperlipidemia and atherosclerosis (57–61), which can increase the risk of diabetes and its complications (62, 63). Diabetes often leads to hyperlipidemia, which increases the risk of atherosclerotic vascular disease, characterized by arterial lesions affected from the intima, usually preceded by accumulation of lipids and complex sugars, hemorrhage and thrombosis, followed by fibrous tissue proliferation and calcium deposition, as well as progressive chemosis and calcification of the arterial middle layer, leading to thickening and sclerosis of the arterial wall and narrowing of the lumen (64, 65). DKD and DR are common result of hyperglycemia-induced accumulation of AGEs, which is inextricably linked to microangiopathy (66, 67). Abnormal thyroid hormone secretion not only directly disrupts endothelial function, but also exacerbates the damage to endothelial cells by the hyperglycemic state, thus contributing to the development of DKD and DR. In addition, abnormal thyroid hormone secretion decreases endothelial nitric oxide availability, which further promotes microvascular damage in diabetic patients (68, 69). Thus, thyroid hormones protect the endothelium of diabetic microvessels from degeneration, which may be reliable evidence to support our main results.

This study exhibits several strengths that warrant investigation. Firstly, the research employed MR methods to evaluate gene-based causality of FT4, TSH, TPOAB, and FT3:FT4 in DKD and DR. MR analysis provides a robust estimate of causality by minimizing reverse causal effects or confounding factors. Secondly, the study used eGFR and ACR as indicators for DKD evaluation, which strengthens the causal relationship between thyroid function and renal function in diabetic patients. Thirdly, the two-sample MR approach was utilized to assess the genetic association between thyroid function and diabetic microvascular complications from various perspectives. Fourthly, the study used multiple sensitivity analyses, such as the simple median, weighted median, and MR-Egger methods, to ensure consistent and robust causal estimation. Finally, the aggregated statistics of the GWAS were collected from European populations, providing a larger sample size than epidemiological studies, and suggesting a more reliable cause-and-effect relationship. Despite meeting the 3 core assumptions, there are still limitations to our MR study. Unobserved pleiotropy, beyond vertical pleiotropy, may exist. Additionally, the lack of an available FT3 instrument limits causal evidence for a genetic association with DKD and DR. Finally, race-based findings may limit generalizability to other populations. Further clinical studies with larger samples are needed to validate these issues.

In conclusion, our study provides direct evidence supporting that genetically based high TSH levels are associated with low eGFR and high DKD risk in diabetic patients, and that ACR in diabetic patients is negatively correlated with FT4 in the reference range and positively correlated with FT3:FT4. We found no genetic evidence of thyroid function associated with DR. These findings suggest that maintaining normal thyroid function and regulation of thyroid hormone secretion may be effective in preventing microvascular complications in diabetes, particularly DKD. Further larger population-based studies are necessary to investigate the causal relationship between thyroid function and diabetic microangiopathy.

## Data availability statement

The original contributions presented in the study are included in the article/**Supplementary Material**. Further inquiries can be directed to the corresponding author.

## Author contributions

HL and ML conceived and designed the study. SD and AD performed the initial data source acquisition. HL examined the data and performed the data analysis. SZ made methodological recommendations for the article. HL wrote the initial manuscript and MZ checked the manuscript and approved the final manuscript. All listed authors made substantial contributions to the manuscript. All authors contributed to the article and approved the submitted version.
