# Supplementary material for: Assessment of the association between genetic factors regulating thyroid function and microvascular complications in diabetes: A two-sample Mendelian randomization study in the European population
Source: Front Endocrinol (Lausanne). 2023 Feb 28;14:1126339. doi: 10.3389/fendo.2023.1126339 (PMC10011638; doi:10.3389/fendo.2023.1126339)
Supplement: Supplementary file 1 [file DataSheet_1.pdf]

Supplementary Online Content

eTable 1. Published associations between associated phenotypes of FT4 and FT3/FT4 and DKD in two-sample MR
eTable 2. Published associations between associated phenotypes of TSH and DKD in two-sample MR
eTable 3. Published associations between associated phenotypes of TPOAb and DKD in two-sample MR
eTable 4. Published associations between associated phenotypes of FT4 and FT3/FT4 and DR in two-sample MR
eTable 5. Published associations between associated phenotypes of TSH and DR in two-sample MR
eTable 6. Published associations between associated phenotypes of TPOAb and DR in two-sample MR
eTable 7. Published associations between associated phenotypes of FT4 and FT3/FT4 and eGFR of people with diabetes in two-sample MR
eTable 8. Published associations between associated phenotypes of TSH and eGFR of people with diabetes in two-sample MR
eTable 9. Published associations between associated phenotypes of TPOAb and eGFR of people with diabetes in two-sample MR
eTable 10. Published associations between associated phenotypes of FT4 and FT3/FT4 and ACR of people with diabetes in two-sample MR
eTable 11. Published associations between associated phenotypes of TSH and ACR of people with diabetes in two-sample MR
eTable 12. Published associations between associated phenotypes of TPOAb and ACR of people with diabetes in two-sample MR
eTable 13. Published associations between associated phenotypes of FT4 and FT3/FT4 and PDR in two-sample MR
eTable 14. Published associations between associated phenotypes of TSH and PDR in two-sample MR
eTable 15. Published associations between associated phenotypes of TPOAb and PDR in two-sample MR
eFigure 1. FT4-associated SNPs with risk of DKD (A. Scatter plot; B. Forest plot; C. Funnel plot; D. Leave-one-out plot)
eFigure 2.TSH-associated SNPs with risk of DKD (A. Scatter plot; B. Forest plot; C. Funnel plot; D. Leave-one-out plot)
eFigure 3.TPOAb-associated SNPs with risk of DKD (A. Scatter plot; B. Forest plot; C. Funnel plot; D. Leave-one-out plot)
eFigure 4.FT4-associated SNPs with risk of DR (A. Scatter plot; B. Forest plot; C. Funnel plot; D. Leave-one-out plot)
eFigure 5.TSH-associated SNPs with risk of DR (A. Scatter plot; B. Forest plot; C. Funnel plot; D. Leave-one-out plot)
eFigure 6.TPOAb-associated SNPs with risk of DR (A. Scatter plot; B. Forest plot; C. Funnel plot; D. Leave-one-out plot)
eFigure 7.FT4-associated SNPs with risk of eGFR (A. Scatter plot; B. Forest plot; C. Funnel plot; D. Leave-one-out plot)
eFigure 8.TSH-associated SNPs with risk of eGFR (A. Scatter plot; B. Forest plot; C. Funnel plot; D. Leave-one-out plot)
eFigure 9.TPOAb-associated SNPs with risk of eGFR (A. Scatter plot; B. Forest plot; C. Funnel plot; D. Leave-one-out plot)
eFigure 10. FT4-associated SNPs with risk of ACR (A. Scatter plot; B. Forest plot; C. Funnel plot; D. Leave-one-out plot)
eFigure 11. TSH-associated SNPs with risk of ACR (A. Scatter plot; B. Forest plot; C. Funnel plot; D. Leave-one-out plot)
eFigure 12. TPOAb-associated SNPs with risk of ACR (A. Scatter plot; B. Forest plot; C. Funnel plot; D. Leave-one-out plot)
eFigure 13. FT4-associated SNPs with risk of PDR (A. Scatter plot; B. Forest plot; C. Funnel plot; D. Leave-one-out plot)
eFigure 14. TSH-associated SNPs with risk of PDR (A. Scatter plot; B. Forest plot; C. Funnel plot; D. Leave-one-out plot)
eFigure 15. TPOAb-associated SNPs with risk of PDR (A. Scatter plot; B. Forest plot; C. Funnel plot; D. Leave-one-out plot)

eTable 1. Published associations between associated phenotypes of FT4 and FT3/FT4 and DKD in two-sample MR

Gene	SNP	Exposure	Chr	EA	OA	EAF	GX	GX(SE)	p-value	F-statistic	GY	GY(SE)	p-value
GLIS3	rs10119187	FT4	9	T	C	0.81	0.048	0.008	8.00E-10	36	-0.014	0.036	0.710
FOXE1	rs10739496	FT4	9	T	C	0.66	0.078	0.007	4.20E-30	124	-0.037	0.027	0.167
NEK6	rs10818937	FT4	9	C	T	0.68	0.039	0.006	4.90E-11	42	-0.024	0.027	0.376
ID4	rs10946313	FT4	6	T	C	0.63	0.044	0.006	6.20E-12	54	-0.016	0.026	0.548
SLC25A52	rs113107469	FT4	18	T	C	0.03	0.200	0.022	1.00E-19	83	0.061	0.091	0.508
LOC728012	rs17185536	FT4	6	T	C	0.24	0.071	0.008	2.70E-20	79	-0.002	0.032	0.957
DIO1	rs2235544	FT4	1	A	C	0.52	0.139	0.007	4.20E-101	394	0.023	0.026	0.372
DIO2	rs225014	FT4	14	T	C	0.64	0.047	0.006	4.60E-17	61	0.012	0.029	0.679
SLCO1B1	rs4149056	FT4	12	C	T	0.16	0.048	0.007	6.30E-11	47	-0.074	0.032	0.021
LHX3	rs4842131	FT4	9	C	T	0.55	0.104	0.008	7.70E-44	169	-0.018	0.026	0.489
ACMSD	rs4954192	FT4	2	C	T	0.57	0.033	0.006	9.30E-09	30	0.009	0.026	0.739
MC4R	rs56069042	FT4	18	A	G	0.95	0.099	0.017	3.60E-09	34	-0.076	0.064	0.237
CA8	rs67583169	FT4	8	C	G	0.86	0.062	0.009	7.10E-12	47	-0.060	0.031	0.051
SOX2-OT	rs6785807	FT4	3	G	A	0.85	0.057	0.009	6.90E-11	40	-0.013	0.029	0.646
SNX29	rs8063103	FT4	16	G	C	0.15	0.051	0.009	7.80E-09	32	-0.052	0.041	0.212
SLC17A4	rs9356988	FT4	6	G	A	0.73	0.052	0.007	5.70E-14	55	-0.044	0.027	0.104
DIO1	rs2235544	FT3:FT4 Ratio	1	C	A	0.49	0.073	0.016	3.60E-13	21	-0.023	0.026	0.371

DKD: diabetic kidney disease. FT4: free thyroxine. FT3: free triiodothyronine. EA: effect allele. OA: other allele. EAF: EA frequency. GX: beta-exposure. GX(SE): standard error of GX. GY: beta-outcome. GY(SE): standard error of GY. References in reference list: Teumer[1] and Panicker[2] .

eTable 2. Published associations between associated phenotypes of TSH and DKD in two-sample MR

Gene	SNP	Exposure	Chr	EA	OA	EAF	GX	GX(SE)	p-value	F-statistic	GY	GY(SE)	p-value
SOX9	rs1042673	TSH	17	A	G	0.520	-0.055	0.006	3.60E-19	84	-0.005	0.052	0.927
ADCY9	rs1045476	TSH	16	A	G	0.180	0.049	0.008	2.40E-09	38	0.060	0.067	0.371
PDE10A	rs1079418	TSH	6	A	G	0.690	0.101	0.007	8.20E-53	208	0.120	0.056	0.032
GLIS3	rs10814915	TSH	9	T	C	0.440	0.042	0.006	5.10E-12	49	0.058	0.052	0.267
CAPZB	rs10917469	TSH	1	A	G	0.840	0.111	0.009	4.00E-39	152	-0.100	0.071	0.157
SULF1	rs10957494	TSH	8	A	G	0.690	-0.040	0.007	1.10E-09	33	0.010	0.057	0.855
TSHR	rs11159482	TSH	14	T	C	0.090	0.085	0.013	6.30E-11	43	0.090	0.111	0.416
GATA3	rs11255790	TSH	10	T	C	0.300	-0.041	0.007	6.80E-10	34	-0.071	0.057	0.212
BCAS3	rs1157994	TSH	17	A	G	0.050	-0.090	0.016	5.30E-09	32	-0.026	0.125	0.837
NR3C2	rs11732089	TSH	4	T	C	0.800	0.115	0.008	1.70E-51	207	0.088	0.064	0.166
TG	rs118039499	TSH	8	A	C	0.980	0.184	0.024	2.00E-14	59	0.075	0.164	0.649
FOXA2	rs1203944	TSH	20	T	C	0.230	-0.051	0.007	2.40E-12	53	0.175	0.063	0.005
PRDM11	rs12284404	TSH	11	A	G	0.270	-0.067	0.007	2.50E-22	92	-0.047	0.056	0.405
PSORS1C1	rs1265091	TSH	6	T	C	0.200	0.057	0.009	3.20E-11	40	0.138	0.066	0.036
TSHR	rs12893151	TSH	14	A	C	0.220	-0.062	0.008	1.00E-15	60	-0.087	0.064	0.173
IGFBP5	rs13015993	TSH	2	A	G	0.730	0.082	0.007	4.50E-32	137	0.002	0.058	0.979
DET1	rs13329353	TSH	15	T	C	0.680	0.061	0.007	5.20E-21	76	0.095	0.055	0.085
SYN2	rs1663070	TSH	3	T	C	0.740	-0.046	0.007	3.50E-11	43	-0.024	0.059	0.683
VAV3	rs17020122	TSH	1	T	C	0.090	0.104	0.011	5.30E-20	89	0.081	0.090	0.367
FAM227B/FGF7	rs17477923	TSH	15	T	C	0.740	0.083	0.007	2.60E-33	141	-0.077	0.059	0.198
MAF	rs17767491	TSH	16	A	G	0.680	0.088	0.007	3.40E-42	158	0.244	0.055	0.000
PDE8B	rs2127387	TSH	5	A	G	0.410	0.144	0.006	1.10E-117	576	0.066	0.053	0.212
NRG1	rs2439301	TSH	8	A	G	0.230	-0.059	0.008	8.20E-15	54	-0.031	0.062	0.614
TM4SF4	rs28502438	TSH	3	T	C	0.570	0.034	0.006	3.70E-08	32	0.077	0.052	0.141

MIR365A	rs30227	TSH	16	T	C	0.610	-0.047	0.006	7.60E-14	61	0.063	0.053	0.230
NFIA	rs334725	TSH	1	A	G	0.950	0.174	0.015	2.40E-32	135	-0.066	0.124	0.593
MBIP	rs398745	TSH	14	A	C	0.590	-0.052	0.006	4.00E-17	75	0.122	0.052	0.020
CADM1	rs4445669	TSH	11	T	C	0.460	-0.040	0.006	5.80E-11	44	0.135	0.052	0.009
INSR	rs4804413	TSH	19	T	C	0.440	0.053	0.006	8.60E-18	78	-0.041	0.052	0.435
PTEN	rs4933466	TSH	10	A	G	0.600	0.040	0.006	5.10E-10	44	-0.023	0.053	0.662
HES1	rs59381142	TSH	3	A	G	0.240	-0.058	0.008	1.70E-14	53	-0.105	0.060	0.082
SPATA13	rs7329958	TSH	13	T	C	0.350	-0.044	0.007	1.10E-11	40	0.090	0.054	0.095
VEGFA/LOC100132354	rs744103	TSH	6	A	T	0.690	0.092	0.007	6.70E-41	173	0.014	0.056	0.800
ITPK1	rs8015085	TSH	14	A	G	0.210	0.067	0.008	2.40E-18	70	0.089	0.065	0.171
ABO	rs8176645	TSH	9	A	T	0.340	0.052	0.006	3.90E-16	75	-0.166	0.064	0.009
C9orf92	rs9298749	TSH	9	A	C	0.590	-0.039	0.006	8.80E-10	42	-0.094	0.054	0.083
VEGFA/LOC100132354	rs9381266	TSH	6	T	C	0.740	0.073	0.007	1.80E-25	109	0.009	0.060	0.880
SASH1	rs9497965	TSH	6	T	C	0.400	0.044	0.006	9.80E-13	54	0.066	0.052	0.210
SOX9	rs963384	TSH	17	T	C	0.460	0.035	0.006	2.80E-08	34	0.046	0.052	0.371

DKD: diabetic kidney disease. TSH: thyroid-stimulating hormone. EA: effect allele. OA: other allele. EAF: EA frequency. GX: beta-exposure. GX(SE): standard error of GX. GY: beta-outcome. GY(SE): standard error of GY. References in reference list: Teumer[1] .

eTable 3. Published associations between associated phenotypes of TPOAb and DKD in two-sample MR

Gene	SNP	Exposure	Chr	EA	OA	EAF	GX	GX(SE)	p-value	F-statistic	GY	GY(SE)	p-value
TPO	rs11675434	TPOAb	17	T	C	0.390	0.020	0.005	7.40E-13	19	-0.008	0.026	0.622
MAGI3	rs1230666	TPOAb	16	A	G	0.160	0.027	0.006	1.80E-09	10	0.184	0.034	0.006
ATXN2,[SH2B3]	rs653178	TPOAb	6	C	T	0.500	0.015	0.004	3.50E-10	11	0.061	0.026	0.001
HLA-DPB1	rs9277555	TPOAb	9	G	A	0.740	0.020	0.005	3.90E-09	15	-0.045	0.030	0.019

DKD: diabetic kidney disease. TPOAb: thyroid peroxidase antibodies. EA: effect allele. OA: other allele. EAF: EA frequency. GX: beta-exposure. GX(SE): standard error of GX. GY: beta-outcome. GY(SE): standard error of GY. References in reference list: Schultheiss[3] and Medici[4] .

eTable 4. Published associations between associated phenotypes of FT4 and FT3/FT4 and DR in two-sample MR

Gene	SNP	Exposure	Chr	EA	OA	EAF	GX	GX(SE)	p-value	F-statistic	GY	GY(SE)	p-value
GLIS3	rs10119187	FT4	9	T	C	0.81	0.048	0.008	8.00E-10	36	-0.006	0.018	0.757
FOXE1	rs10739496	FT4	9	T	C	0.66	0.078	0.007	4.20E-30	124	0.004	0.014	0.748
NEK6	rs10818937	FT4	9	C	T	0.68	0.039	0.006	4.90E-11	42	0.004	0.014	0.797
ID4	rs10946313	FT4	6	T	C	0.63	0.044	0.006	6.20E-12	54	0.012	0.013	0.353
SLC25A52	rs113107469	FT4	18	T	C	0.03	0.200	0.022	1.00E-19	83	0.032	0.046	0.491
LOC728012	rs17185536	FT4	6	T	C	0.24	0.071	0.008	2.70E-20	79	0.002	0.016	0.892
DIO1	rs2235544	FT4	1	A	C	0.52	0.139	0.007	4.20E-101	394	-0.003	0.013	0.797
DIO2	rs225014	FT4	14	T	C	0.64	0.047	0.006	4.60E-17	61	0.007	0.014	0.610
SLCO1B1	rs4149056	FT4	12	C	T	0.16	0.048	0.007	6.30E-11	47	-0.036	0.016	0.023
LHX3	rs4842131	FT4	9	C	T	0.55	0.104	0.008	7.70E-44	169	-0.009	0.013	0.474
ACMSD	rs4954192	FT4	2	C	T	0.57	0.033	0.006	9.30E-09	30	0.001	0.013	0.923
MC4R	rs56069042	FT4	18	A	G	0.95	0.099	0.017	3.60E-09	34	0.022	0.032	0.497
CA8	rs67583169	FT4	8	C	G	0.86	0.062	0.009	7.10E-12	47	-0.022	0.016	0.155
SOX2-OT	rs6785807	FT4	3	G	A	0.85	0.057	0.009	6.90E-11	40	0.022	0.015	0.137
SNX29	rs8063103	FT4	16	G	C	0.15	0.051	0.009	7.80E-09	32	-0.001	0.021	0.972
SLC17A4	rs9356988	FT4	6	G	A	0.73	0.052	0.007	5.70E-14	55	-0.053	0.013	0.000
DIO1	rs2235544	FT3:FT4 Ratio	1	C	A	0.49	0.073	0.016	3.60E-13	21	0.003	0.013	0.800

DR: diabetic retinopathy. FT4: free thyroxine. FT3: free triiodothyronine. EA: effect allele. OA: other allele. EAF: EA frequency. GX: beta-exposure. GX(SE): standard error of GX. GY: beta-outcome. GY(SE): standard error of GY. References in reference list: Teumer[1] and Panicker[2] .

eTable 5. Published associations between associated phenotypes of TSH and DR in two-sample MR

Gene	SNP	Exposure	Chr	EA	OA	EAF	GX	GX(SE)	p-value	F-statistic	GY	GY(SE)	p-value
SOX9	rs1042673	TSH	17	A	G	0.520	-0.055	0.006	3.60E-19	84	0.009	0.013	0.505
ADCY9	rs1045476	TSH	16	A	G	0.180	0.049	0.008	2.40E-09	38	0.011	0.017	0.520
PDE10A	rs1079418	TSH	6	A	G	0.690	0.101	0.007	8.20E-53	208	-0.018	0.015	0.230
GLIS3	rs10814915	TSH	9	T	C	0.440	0.042	0.006	5.10E-12	49	0.024	0.013	0.063
CAPZB	rs10917469	TSH	1	A	G	0.840	0.111	0.009	4.00E-39	152	0.019	0.020	0.338
SULF1	rs10957494	TSH	8	A	G	0.690	-0.040	0.007	1.10E-09	33	-0.022	0.014	0.119
TSHR	rs11159482	TSH	14	T	C	0.090	0.085	0.013	6.30E-11	43	0.014	0.026	0.604
GATA3	rs11255790	TSH	10	T	C	0.300	-0.041	0.007	6.80E-10	34	-0.006	0.017	0.733
BCAS3	rs1157994	TSH	17	A	G	0.050	-0.090	0.016	5.30E-09	32	-0.059	0.032	0.064
NR3C2	rs11732089	TSH	4	T	C	0.800	0.115	0.008	1.70E-51	207	-0.027	0.020	0.171
FOXA2	rs1203944	TSH	20	T	C	0.230	-0.051	0.007	2.40E-12	53	-0.015	0.016	0.358
PRDM11	rs12284404	TSH	11	A	G	0.270	-0.067	0.007	2.50E-22	92	-0.006	0.014	0.665
PSORS1C1	rs1265091	TSH	6	T	C	0.200	0.057	0.009	3.20E-11	40	0.017	0.019	0.387
TSHR	rs12893151	TSH	14	A	C	0.220	-0.062	0.008	1.00E-15	60	0.005	0.015	0.746
IGFBP5	rs13015993	TSH	2	A	G	0.730	0.082	0.007	4.50E-32	137	0.002	0.013	0.866
DET1	rs13329353	TSH	15	T	C	0.680	0.061	0.007	5.20E-21	76	0.010	0.014	0.471
SYN2	rs1663070	TSH	3	T	C	0.740	-0.046	0.007	3.50E-11	43	-0.014	0.015	0.372
VAV3	rs17020122	TSH	1	T	C	0.090	0.104	0.011	5.30E-20	89	-0.012	0.020	0.545
FAM227B/FGF7	rs17477923	TSH	15	T	C	0.740	0.083	0.007	2.60E-33	141	-0.006	0.014	0.644
MAF	rs17767491	TSH	16	A	G	0.680	0.088	0.007	3.40E-42	158	0.013	0.014	0.350
PDE8B	rs2127387	TSH	5	A	G	0.410	0.144	0.006	1.10E-117	576	0.004	0.013	0.762
NRG1	rs2439301	TSH	8	A	G	0.230	-0.059	0.008	8.20E-15	54	-0.005	0.015	0.737
TM4SF4	rs28502438	TSH	3	T	C	0.570	0.034	0.006	3.70E-08	32	0.003	0.013	0.843
MIR365A	rs30227	TSH	16	T	C	0.610	-0.047	0.006	7.60E-14	61	0.022	0.013	0.098

NFIA	rs334725	TSH	1	A	G	0.950	0.174	0.015	2.40E-32	135	0.014	0.046	0.767
MBIP	rs398745	TSH	14	A	C	0.590	-0.052	0.006	4.00E-17	75	0.014	0.013	0.299
CADM1	rs4445669	TSH	11	T	C	0.460	-0.040	0.006	5.80E-11	44	-0.013	0.013	0.327
INSR	rs4804413	TSH	19	T	C	0.440	0.053	0.006	8.60E-18	78	0.004	0.013	0.762
PTEN	rs4933466	TSH	10	A	G	0.600	0.040	0.006	5.10E-10	44	-0.004	0.013	0.746
HES1	rs59381142	TSH	3	A	G	0.240	-0.058	0.008	1.70E-14	53	0.006	0.017	0.733
SPATA13	rs7329958	TSH	13	T	C	0.350	-0.044	0.007	1.10E-11	40	0.014	0.014	0.295
VEGFA/LOC100132354	rs744103	TSH	6	A	T	0.690	0.092	0.007	6.70E-41	173	-0.012	0.014	0.389
ITPK1	rs8015085	TSH	14	A	G	0.210	0.067	0.008	2.40E-18	70	-0.037	0.017	0.026
C9orf92	rs9298749	TSH	9	A	C	0.590	-0.039	0.006	8.80E-10	42	0.027	0.013	0.042
VEGFA/LOC100132354	rs9381266	TSH	6	T	C	0.740	0.073	0.007	1.80E-25	109	-0.007	0.016	0.665
SASH1	rs9497965	TSH	6	T	C	0.400	0.044	0.006	9.80E-13	54	0.030	0.014	0.028
SOX9	rs963384	TSH	17	T	C	0.460	0.035	0.006	2.80E-08	34	-0.029	0.013	0.023

DR: diabetic retinopathy. TSH: thyroid-stimulating hormone. EA: effect allele. OA: other allele. EAF: EA frequency. GX: beta-exposure. GX(SE): standard error of GX. GY: beta-outcome. GY(SE): standard error of GY. References in reference list: Teumer[1] .

eTable 6. Published associations between associated phenotypes of TPOAb and DR in two-sample MR

Gene	SNP	Exposure	Chr	EA	OA	EAF	GX	GX(SE)	p-value	F-statistic	GY	GY(SE)	p-value
TPO	rs11675434	TPOAb	17	T	C	0.390	0.020	0.005	7.40E-13	19	-0.007	0.013	0.604
MAGI3	rs1230666	TPOAb	16	A	G	0.160	0.027	0.006	1.80E-09	10	0.118	0.017	0.000
ATXN2,[SH2B3]	rs653178	TPOAb	6	C	T	0.500	0.015	0.004	3.50E-10	11	0.051	0.013	0.000
HLA-DPB1	rs9277555	TPOAb	9	G	A	0.740	0.020	0.005	3.90E-09	15	-0.037	0.015	0.015

DR: diabetic retinopathy. TPOAb: thyroid peroxidase antibodies. EA: effect allele. OA: other allele. EAF: EA frequency. GX: beta-exposure. GX(SE): standard error of GX. GY: beta-outcome. GY(SE): standard error of GY. References in reference list: Schultheiss[3] and Medici[4] .

eTable 7. Published associations between associated phenotypes of FT4 and FT3/FT4 and eGFR of people with diabetes in two-sample MR

Gene	SNP	Exposure	Chr	EA	OA	EAF	GX	GX(SE)	p-value	F-statistic	GY	GY(SE)	p-value
FOXE1	rs10739496	FT4	9	T	C	0.633	0.078	0.007	4.20E-30	124	0.001	0.004	0.710
NEK6	rs10818937	FT4	9	C	T	0.715	0.039	0.006	4.90E-11	42	0.003	0.004	0.470
ID4	rs10946313	FT4	6	T	C	0.584	0.044	0.006	6.20E-12	54	-0.001	0.004	0.870
LOC728012	rs17185536	FT4	6	T	C	0.273	0.071	0.008	2.70E-20	79	0.008	0.004	0.070
DIO1	rs2235544	FT4	1	A	C	0.544	0.139	0.007	4.20E-101	394	0.002	0.004	0.540
DIO2	rs225014	FT4	14	T	C	0.611	0.047	0.006	4.60E-17	61	-0.004	0.004	0.280
SLCO1B1	rs4149056	FT4	12	C	T	0.150	0.048	0.007	6.30E-11	47	0.009	0.005	0.060
ACMSD	rs4954192	FT4	2	C	T	0.643	0.033	0.006	9.30E-09	30	0.007	0.004	0.074
SOX2-OT	rs6785807	FT4	3	G	A	0.867	0.057	0.009	6.90E-11	40	-0.001	0.005	0.820
SLC17A4	rs9356988	FT4	6	G	A	0.754	0.052	0.007	5.70E-14	55	-0.002	0.004	0.630
DIO1	rs2235544	FT3:FT4 Ratio	1	C	A	0.49	0.073	0.016	3.60E-13	21	0.002	0.004	0.540

eGFR: estimated glomerular filtration rate. FT4: free thyroxine. FT3: free triiodothyronine. EA: effect allele. OA: other allele. EAF: EA frequency. GX: beta-exposure. GX(SE): standard error of GX. GY: beta-outcome. GY(SE): standard error of GY. References in reference list: Teumer[1] and Panicker[2] .

eTable 8. Published associations between associated phenotypes of TSH and eGFR of people with diabetes in two-sample MR

Gene	SNP	Exposure	Chr	EA	OA	EAF	GX	GX(SE)	p-value	F-statistic	GY	GY(SE)	p-value
PDE10A	rs1079418	TSH	6	A	G	0.690	0.101	0.007	8.20E-53	208	0.004	0.004	0.370
CAPZB	rs10917469	TSH	1	A	G	0.840	0.111	0.009	4.00E-39	152	0.002	0.005	0.690
GATA3	rs11255790	TSH	10	T	C	0.300	-0.041	0.007	6.80E-10	34	0.003	0.004	0.520
NR3C2	rs11732089	TSH	4	T	C	0.800	0.115	0.008	1.70E-51	207	-0.004	0.004	0.370
FOXA2	rs1203944	TSH	20	T	C	0.230	-0.051	0.007	2.40E-12	53	0.003	0.004	0.480
IGFBP5	rs13015993	TSH	2	A	G	0.730	0.082	0.007	4.50E-32	137	0.002	0.004	0.700
DET1	rs13329353	TSH	15	T	C	0.680	0.061	0.007	5.20E-21	76	-0.002	0.004	0.660
SYN2	rs1663070	TSH	3	T	C	0.740	-0.046	0.007	3.50E-11	43	0.012	0.004	0.004
VAV3	rs17020122	TSH	1	T	C	0.090	0.104	0.011	5.30E-20	89	-0.006	0.006	0.380
FAM227B/FGF7	rs17477923	TSH	15	T	C	0.740	0.083	0.007	2.60E-33	141	-0.001	0.004	0.730
MAF	rs17767491	TSH	16	A	G	0.680	0.088	0.007	3.40E-42	158	0.002	0.004	0.610
MIR365A	rs30227	TSH	16	T	C	0.610	-0.047	0.006	7.60E-14	61	-0.003	0.004	0.470
MBIP	rs398745	TSH	14	A	C	0.590	-0.052	0.006	4.00E-17	75	0.002	0.004	0.650
CADM1	rs4445669	TSH	11	T	C	0.460	-0.040	0.006	5.80E-11	44	0.003	0.004	0.410
PTEN	rs4933466	TSH	10	A	G	0.600	0.040	0.006	5.10E-10	44	-0.010	0.004	0.012
VEGFA/LOC100132354	rs744103	TSH	6	A	T	0.690	0.092	0.007	6.70E-41	173	-0.005	0.004	0.180
C9orf92	rs9298749	TSH	9	A	C	0.590	-0.039	0.006	8.80E-10	42	0.000	0.004	0.900
VEGFA/LOC100132354	rs9381266	TSH	6	T	C	0.740	0.073	0.007	1.80E-25	109	-0.013	0.004	0.002
SASH1	rs9497965	TSH	6	T	C	0.400	0.044	0.006	9.80E-13	54	-0.004	0.004	0.250

eGFR: estimated glomerular filtration rate. TSH: thyroid-stimulating hormone. EA: effect allele. OA: other allele. EAF: EA frequency. GX: beta-exposure. GX(SE): standard error of GX. GY: beta-outcome. GY(SE): standard error of GY. References in reference list: Teumer[1] .

eTable 9. Published associations between associated phenotypes of TPOAb and eGFR of people with diabetes in two-sample MR

Gene	SNP	Exposure	Chr	EA	OA	EAF	GX	GX(SE)	p-value	F-statistic	GY	GY(SE)	p-value
TPO	rs11675434	TPOAb	17	T	C	0.390	0.020	0.005	7.40E-13	19	-0.003	0.004	0.350
MAGI3	rs1230666	TPOAb	16	A	G	0.160	0.027	0.006	1.80E-09	10	-0.001	0.005	0.820
ATXN2,[SH2B3]	rs653178	TPOAb	6	C	T	0.500	0.015	0.004	3.50E-10	11	-0.005	0.004	0.160
HLA-DPB1	rs9277555	TPOAb	9	G	A	0.740	0.020	0.005	3.90E-09	15	0.006	0.004	0.150

eGFR: estimated glomerular filtration rate. TPOAb: thyroid peroxidase antibodies. EA: effect allele. OA: other allele. EAF: EA frequency. GX: beta-exposure. GX(SE): standard error of GX. GY: beta-outcome. GY(SE): standard error of GY. References in reference list: Schultheiss[3] and Medici[4] .

eTable 10. Published associations between associated phenotypes of FT4 and FT3/FT4 and ACR of people with diabetes in two-sample MR

Gene	SNP	Exposure	Chr	EA	OA	EAF	GX	GX(SE)	p-value	F-statistic	GY	GY(SE)	p-value
FOXE1	rs10739496	FT4	9	T	C	0.633	0.078	0.007	4.20E-30	124	-0.005	0.006	0.400
NEK6	rs10818937	FT4	9	C	T	0.715	0.039	0.006	4.90E-11	42	0.000	0.006	0.960
ID4	rs10946313	FT4	6	T	C	0.584	0.044	0.006	6.20E-12	54	-0.011	0.007	0.140
LOC728012	rs17185536	FT4	6	T	C	0.273	0.071	0.008	2.70E-20	79	-0.004	0.008	0.640
DIO1	rs2235544	FT4	1	A	C	0.544	0.139	0.007	4.20E-101	394	-0.013	0.007	0.046
DIO2	rs225014	FT4	14	T	C	0.611	0.047	0.006	4.60E-17	61	-0.006	0.006	0.330
SLCO1B1	rs4149056	FT4	12	C	T	0.150	0.048	0.007	6.30E-11	47	-0.003	0.009	0.710
ACMSD	rs4954192	FT4	2	C	T	0.643	0.033	0.006	9.30E-09	30	0.014	0.007	0.033
SOX2-OT	rs6785807	FT4	3	G	A	0.867	0.057	0.009	6.90E-11	40	-0.016	0.010	0.100
SLC17A4	rs9356988	FT4	6	G	A	0.754	0.052	0.007	5.70E-14	55	-0.001	0.007	0.840
DIO1	rs2235544	FT3:FT4 Ratio	1	C	A	0.49	0.073	0.016	3.60E-13	21	0.013	0.007	0.046

ACR: urinary albumin-to-creatinine ratio. FT4: free thyroxine. FT3: free triiodothyronine. EA: effect allele. OA: other allele. EAF: EA frequency. GX: beta-exposure. GX(SE): standard error of GX. GY: beta-outcome. GY(SE): standard error of GY. References in reference list: Teumer[1] and Panicker[2] .

eTable 11. Published associations between associated phenotypes of TSH and ACR of people with diabetes in two-sample MR

Gene	SNP	Exposure	Chr	EA	OA	EAF	GX	GX(SE)	p-value	F-statistic	GY	GY(SE)	p-value
PDE10A	rs1079418	TSH	6	A	G	0.690	0.101	0.007	8.20E-53	208	-0.004	0.008	0.620
CAPZB	rs10917469	TSH	1	A	G	0.840	0.111	0.009	4.00E-39	152	0.006	0.008	0.420
GATA3	rs11255790	TSH	10	T	C	0.300	-0.041	0.007	6.80E-10	34	0.008	0.006	0.210
NR3C2	rs11732089	TSH	4	T	C	0.800	0.115	0.008	1.70E-51	207	-0.007	0.008	0.360
FOXA2	rs1203944	TSH	20	T	C	0.230	-0.051	0.007	2.40E-12	53	-0.003	0.007	0.710
IGFBP5	rs13015993	TSH	2	A	G	0.730	0.082	0.007	4.50E-32	137	0.003	0.007	0.660
DET1	rs13329353	TSH	15	T	C	0.680	0.061	0.007	5.20E-21	76	-0.003	0.006	0.640
SYN2	rs1663070	TSH	3	T	C	0.740	-0.046	0.007	3.50E-11	43	0.009	0.008	0.240
VAV3	rs17020122	TSH	1	T	C	0.090	0.104	0.011	5.30E-20	89	0.002	0.013	0.900
FAM227B/FGF7	rs17477923	TSH	15	T	C	0.740	0.083	0.007	2.60E-33	141	-0.013	0.007	0.061
MAF	rs17767491	TSH	16	A	G	0.680	0.088	0.007	3.40E-42	158	0.001	0.006	0.850
MIR365A	rs30227	TSH	16	T	C	0.610	-0.047	0.006	7.60E-14	61	0.003	0.007	0.710
MBIP	rs398745	TSH	14	A	C	0.590	-0.052	0.006	4.00E-17	75	-0.001	0.006	0.880
CADM1	rs4445669	TSH	11	T	C	0.460	-0.040	0.006	5.80E-11	44	-0.003	0.006	0.630
PTEN	rs4933466	TSH	10	A	G	0.600	0.040	0.006	5.10E-10	44	0.005	0.007	0.520
VEGFA/LOC100132354	rs744103	TSH	6	A	T	0.690	0.092	0.007	6.70E-41	173	-0.019	0.007	0.005
C9orf92	rs9298749	TSH	9	A	C	0.590	-0.039	0.006	8.80E-10	42	0.004	0.007	0.560
VEGFA/LOC100132354	rs9381266	TSH	6	T	C	0.740	0.073	0.007	1.80E-25	109	-0.005	0.008	0.580
SASH1	rs9497965	TSH	6	T	C	0.400	0.044	0.006	9.80E-13	54	0.003	0.007	0.640

ACR: urinary albumin-to-creatinine ratio. TSH: thyroid-stimulating hormone. EA: effect allele. OA: other allele. EAF: EA frequency. GX: beta-exposure. GX(SE): standard error of GX. GY: beta-outcome. GY(SE): standard error of GY. References in reference list: Teumer[1] .

eTable 12. Published associations between associated phenotypes of TPOAb and ACR of people with diabetes in two-sample MR

Gene	SNP	Exposure	Chr	EA	OA	EAF	GX	GX(SE)	p-value	F-statistic	GY	GY(SE)	p-value
TPO	rs11675434	TPOAb	17	T	C	0.390	0.020	0.005	7.40E-13	19	0.010	0.007	0.140
MAGI3	rs1230666	TPOAb	16	A	G	0.160	0.027	0.006	1.80E-09	10	-0.009	0.010	0.350
ATXN2,[SH2B3]	rs653178	TPOAb	6	C	T	0.500	0.015	0.004	3.50E-10	11	0.002	0.007	0.810
HLA-DPB1	rs9277555	TPOAb	9	G	A	0.740	0.020	0.005	3.90E-09	15	-0.001	0.007	0.890

ACR: urinary albumin-to-creatinine ratio. TPOAb: thyroid peroxidase antibodies. EA: effect allele. OA: other allele. EAF: EA frequency. GX: beta-exposure. GX(SE): standard error of GX. GY: beta-outcome. GY(SE): standard error of GY. References in reference list: Schultheiss[3] and Medici[4] .

eTable 13. Published associations between associated phenotypes of FT4 and FT3/FT4 and PDR in two-sample MR

Gene	SNP	Exposure	Chr	EA	OA	EAF	GX	GX(SE)	p-value	F-statistic	GY	GY(SE)	p-value
GLIS3	rs10119187	FT4	9	T	C	0.81	0.048	0.008	8.00E-10	36	-0.009	0.023	0.689
FOXE1	rs10739496	FT4	9	T	C	0.66	0.078	0.007	4.20E-30	124	-0.007	0.017	0.687
NEK6	rs10818937	FT4	9	C	T	0.68	0.039	0.006	4.90E-11	42	-0.002	0.017	0.911
ID4	rs10946313	FT4	6	T	C	0.63	0.044	0.006	6.20E-12	54	-0.006	0.017	0.739
SLC25A52	rs113107469	FT4	18	T	C	0.03	0.200	0.022	1.00E-19	83	0.090	0.059	0.126
LOC728012	rs17185536	FT4	6	T	C	0.24	0.071	0.008	2.70E-20	79	-0.003	0.020	0.895
DIO1	rs2235544	FT4	1	A	C	0.52	0.139	0.007	4.20E-101	394	-0.002	0.017	0.899
DIO2	rs225014	FT4	14	T	C	0.64	0.047	0.006	4.60E-17	61	0.025	0.018	0.166
SLCO1B1	rs4149056	FT4	12	C	T	0.16	0.048	0.007	6.30E-11	47	-0.066	0.021	0.001
LHX3	rs4842131	FT4	9	C	T	0.55	0.104	0.008	7.70E-44	169	-0.007	0.016	0.653
ACMSD	rs4954192	FT4	2	C	T	0.57	0.033	0.006	9.30E-09	30	0.005	0.017	0.767
MC4R	rs56069042	FT4	18	A	G	0.95	0.099	0.017	3.60E-09	34	0.025	0.041	0.551
CA8	rs67583169	FT4	8	C	G	0.86	0.062	0.009	7.10E-12	47	-0.032	0.020	0.106
SOX2-OT	rs6785807	FT4	3	G	A	0.85	0.057	0.009	6.90E-11	40	0.013	0.019	0.495
SNX29	rs8063103	FT4	16	G	C	0.15	0.051	0.009	7.80E-09	32	0.010	0.027	0.695
SLC17A4	rs9356988	FT4	6	G	A	0.73	0.052	0.007	5.70E-14	55	-0.064	0.017	0.000
DIO1	rs2235544	FT3:FT4 Ratio	1	C	A	0.49	0.073	0.016	3.60E-13	21	0.002	0.017	0.899

PDR: proliferative diabetic retinopathy. FT4: free thyroxine. FT3: free triiodothyronine. EA: effect allele. OA: other allele. EAF: EA frequency. GX: beta-exposure. GX(SE): standard error of GX. GY: beta-outcome. GY(SE): standard error of GY. References in reference list: Teumer[1] and Panicker[2] .

eTable 14. Published associations between associated phenotypes of TSH and PDR in two-sample MR

Gene	SNP	Exposure	Chr	EA	OA	EAF	GX	GX(SE)	p-value	F-statistic	GY	GY(SE)	p-value
SOX9	rs1042673	TSH	17	A	G	0.520	-0.055	0.006	3.60E-19	84	-0.004	0.017	0.833
ADCY9	rs1045476	TSH	16	A	G	0.180	0.049	0.008	2.40E-09	38	0.011	0.021	0.619
PDE10A	rs1079418	TSH	6	A	G	0.690	0.101	0.007	8.20E-53	208	-0.041	0.019	0.028
GLIS3	rs10814915	TSH	9	T	C	0.440	0.042	0.006	5.10E-12	49	0.046	0.016	0.005
CAPZB	rs10917469	TSH	1	A	G	0.840	0.111	0.009	4.00E-39	152	0.029	0.025	0.242
SULF1	rs10957494	TSH	8	A	G	0.690	-0.040	0.007	1.10E-09	33	-0.023	0.018	0.196
TSHR	rs11159482	TSH	14	T	C	0.090	0.085	0.013	6.30E-11	43	-0.045	0.034	0.186
GATA3	rs11255790	TSH	10	T	C	0.300	-0.041	0.007	6.80E-10	34	-0.004	0.021	0.837
BCAS3	rs1157994	TSH	17	A	G	0.050	-0.090	0.016	5.30E-09	32	-0.070	0.041	0.085
NR3C2	rs11732089	TSH	4	T	C	0.800	0.115	0.008	1.70E-51	207	-0.041	0.025	0.099
FOXA2	rs1203944	TSH	20	T	C	0.230	-0.051	0.007	2.40E-12	53	0.003	0.020	0.903
PRDM11	rs12284404	TSH	11	A	G	0.270	-0.067	0.007	2.50E-22	92	-0.009	0.018	0.629
PSORS1C1	rs1265091	TSH	6	T	C	0.200	0.057	0.009	3.20E-11	40	0.048	0.024	0.050
TSHR	rs12893151	TSH	14	A	C	0.220	-0.062	0.008	1.00E-15	60	-0.015	0.019	0.421
IGFBP5	rs13015993	TSH	2	A	G	0.730	0.082	0.007	4.50E-32	137	-0.004	0.017	0.812
DET1	rs13329353	TSH	15	T	C	0.680	0.061	0.007	5.20E-21	76	0.022	0.017	0.209
SYN2	rs1663070	TSH	3	T	C	0.740	-0.046	0.007	3.50E-11	43	-0.011	0.020	0.566
VAV3	rs17020122	TSH	1	T	C	0.090	0.104	0.011	5.30E-20	89	-0.020	0.026	0.445
FAM227B/FGF7	rs17477923	TSH	15	T	C	0.740	0.083	0.007	2.60E-33	141	-0.008	0.018	0.656
MAF	rs17767491	TSH	16	A	G	0.680	0.088	0.007	3.40E-42	158	0.010	0.018	0.592
PDE8B	rs2127387	TSH	5	A	G	0.410	0.144	0.006	1.10E-117	576	0.006	0.017	0.708
NRG1	rs2439301	TSH	8	A	G	0.230	-0.059	0.008	8.20E-15	54	0.007	0.019	0.698
TM4SF4	rs28502438	TSH	3	T	C	0.570	0.034	0.006	3.70E-08	32	-0.001	0.017	0.964
MIR365A	rs30227	TSH	16	T	C	0.610	-0.047	0.006	7.60E-14	61	0.022	0.017	0.195

NFIA	rs334725	TSH	1	A	G	0.950	0.174	0.015	2.40E-32	135	-0.032	0.059	0.582
MBIP	rs398745	TSH	14	A	C	0.590	-0.052	0.006	4.00E-17	75	0.016	0.017	0.346
CADM1	rs4445669	TSH	11	T	C	0.460	-0.040	0.006	5.80E-11	44	-0.021	0.017	0.207
INSR	rs4804413	TSH	19	T	C	0.440	0.053	0.006	8.60E-18	78	0.011	0.017	0.511
PTEN	rs4933466	TSH	10	A	G	0.600	0.040	0.006	5.10E-10	44	-0.001	0.017	0.952
HES1	rs59381142	TSH	3	A	G	0.240	-0.058	0.008	1.70E-14	53	0.032	0.021	0.125
SPATA13	rs7329958	TSH	13	T	C	0.350	-0.044	0.007	1.10E-11	40	0.016	0.017	0.348
VEGFA/LOC100132354	rs744103	TSH	6	A	T	0.690	0.092	0.007	6.70E-41	173	-0.017	0.018	0.335
ITPK1	rs8015085	TSH	14	A	G	0.210	0.067	0.008	2.40E-18	70	-0.022	0.021	0.299
C9orf92	rs9298749	TSH	9	A	C	0.590	-0.039	0.006	8.80E-10	42	0.026	0.017	0.120
VEGFA/LOC100132354	rs9381266	TSH	6	T	C	0.740	0.073	0.007	1.80E-25	109	-0.028	0.020	0.167
SASH1	rs9497965	TSH	6	T	C	0.400	0.044	0.006	9.80E-13	54	0.036	0.017	0.037
SOX9	rs963384	TSH	17	T	C	0.460	0.035	0.006	2.80E-08	34	-0.043	0.017	0.010

PDR: proliferative diabetic retinopathy. TSH: thyroid-stimulating hormone. EA: effect allele. OA: other allele. EAF: EA frequency. GX: beta-exposure. GX(SE): standard error of GX. GY: beta-outcome. GY(SE): standard error of GY. References in reference list: Teumer[1] .

eTable 15. Published associations between associated phenotypes of TPOAb and PDR in two-sample MR

Gene	SNP	Exposure	Chr	EA	OA	EAF	GX	GX(SE)	p-value	F-statistic	GY	GY(SE)	p-value
TPO	rs11675434	TPOAb	17	T	C	0.390	0.020	0.005	7.40E-13	19	-0.008	0.017	0.623
MAGI3	rs1230666	TPOAb	16	A	G	0.160	0.027	0.006	1.80E-09	10	0.184	0.022	0.000
ATXN2,[SH2B3]	rs653178	TPOAb	6	C	T	0.500	0.015	0.004	3.50E-10	11	0.061	0.017	0.000
HLA-DPB1	rs9277555	TPOAb	9	G	A	0.740	0.020	0.005	3.90E-09	15	-0.046	0.019	0.019

PDR: proliferative diabetic retinopathy. TPOAb: thyroid peroxidase antibodies. EA: effect allele. OA: other allele. EAF: EA frequency. GX: beta-exposure. GX(SE): standard error of GX. GY: beta-outcome. GY(SE): standard error of GY. References in reference list: Schultheiss[3] and Medici[4] .

eFigure 1. FT4-associated SNPs with risk of DKD

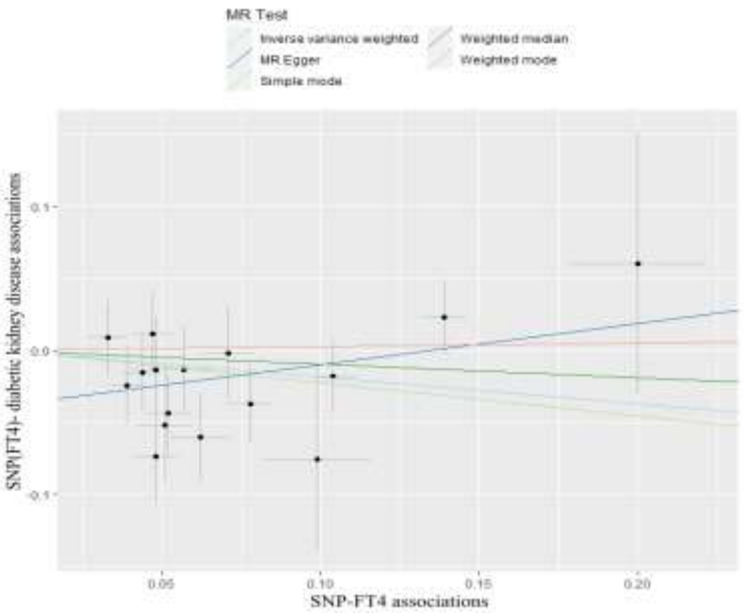

A. Scatter plot of causal effect of FT4 on DKD

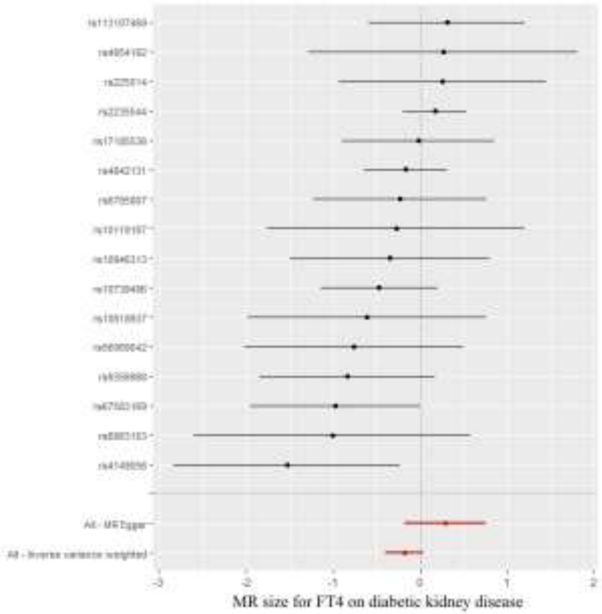

B. Forest plot of causal effect of FT4 on DKD

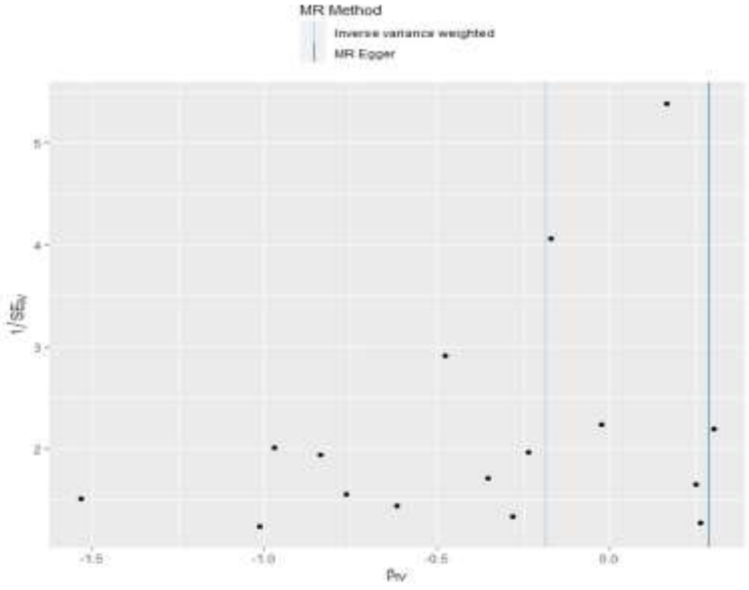

C. Funnel plot of causal effect of FT4 on DKD

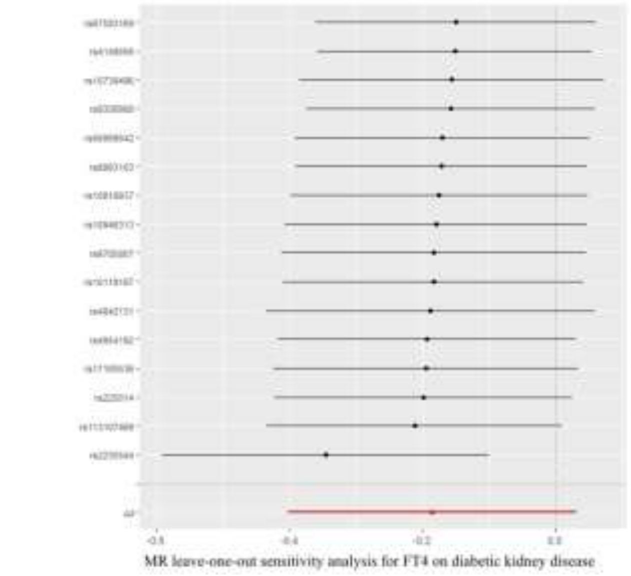

D. Leave-one-out analysis for IVW-RE MR of FT4 on DKD

eFigure 2. TSH-associated SNPs with risk of DKD

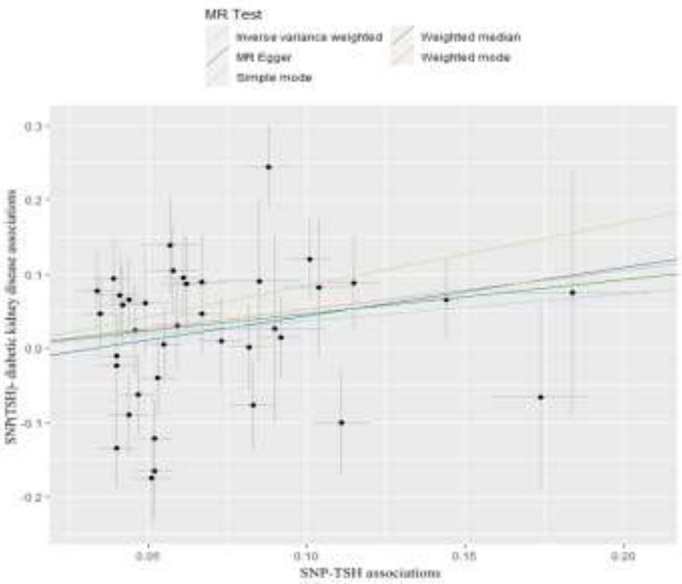

A. Scatter plot of causal effect of TSH on DKD

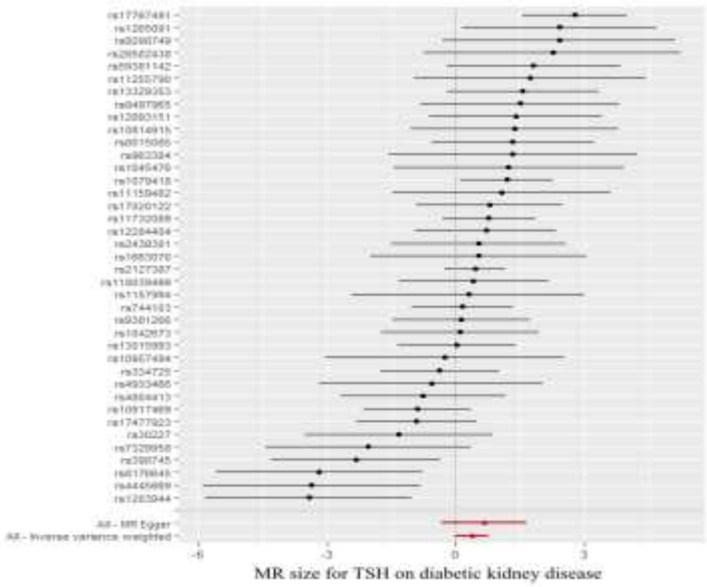

B. Forest plot of causal effect of TSH on DKD

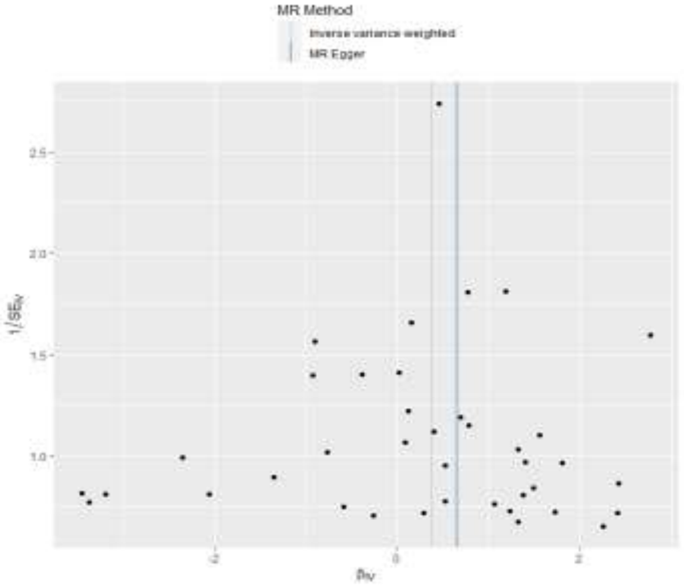

C. Funnel plot of causal effect of TSH on DKD

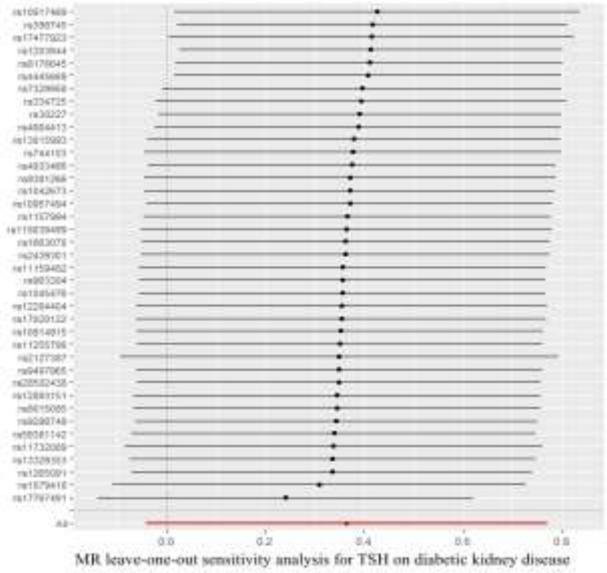

D. Leave-one-out analysis for IVW-RE MR of TSH on DKD

eFigure 3. TPOAb-associated SNPs with risk of DKD

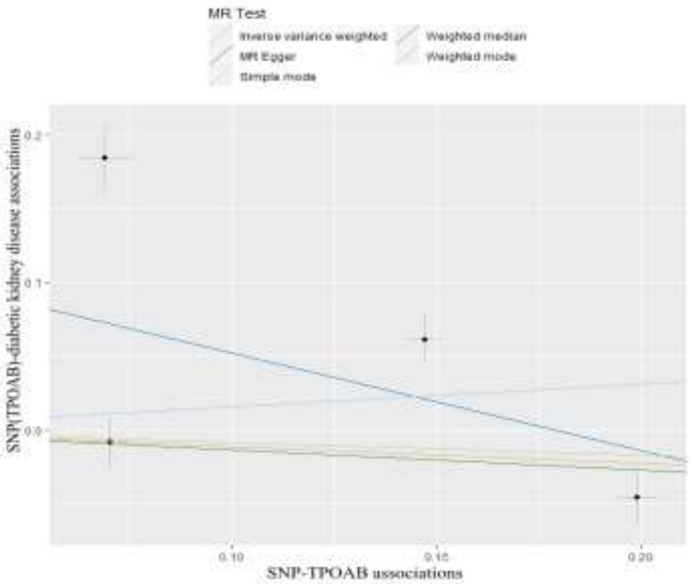

A. Scatter plot of causal effect of TPOAb on DKD

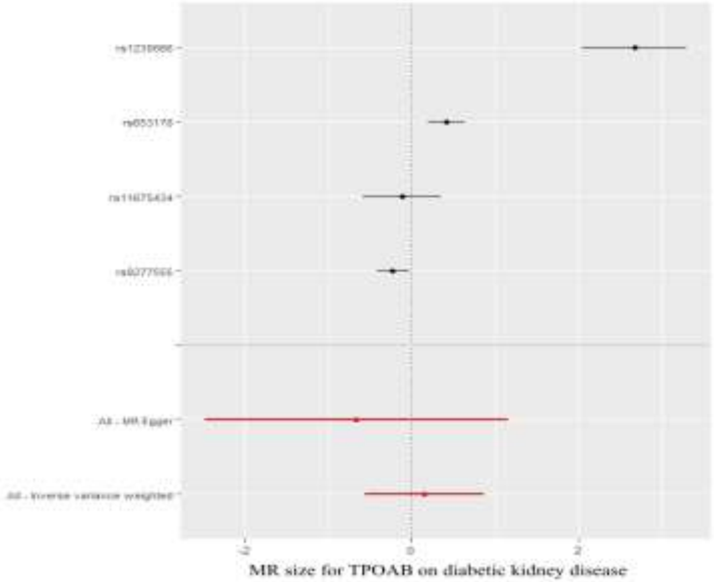

B. Forest plot of causal effect of TPOAb on DKD

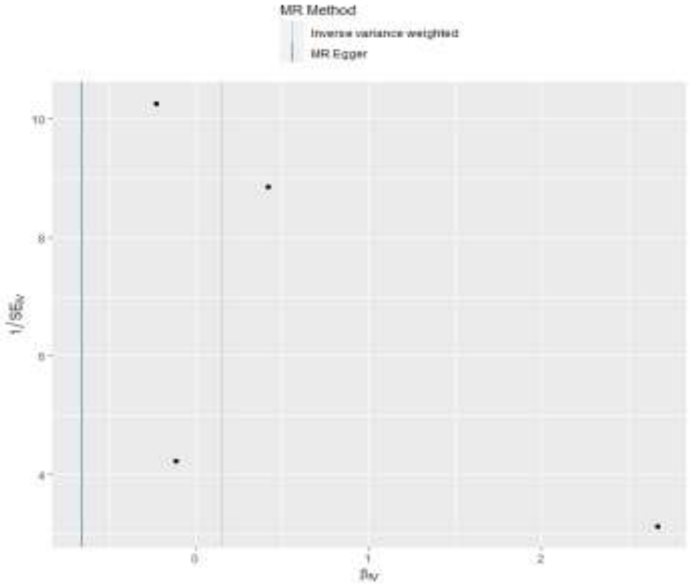

C. Funnel plot of causal effect of TPOAb on DKD

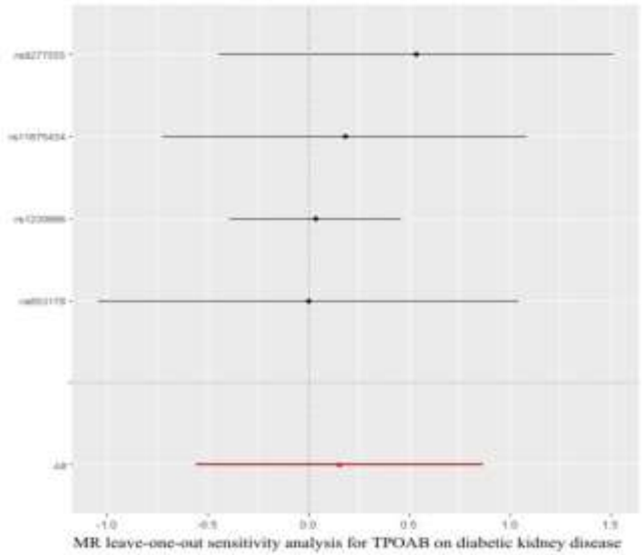

D. Leave-one-out analysis for IVW-RE MR of TPOAb on DKD

eFigure 4. FT4-associated SNPs with risk of DR

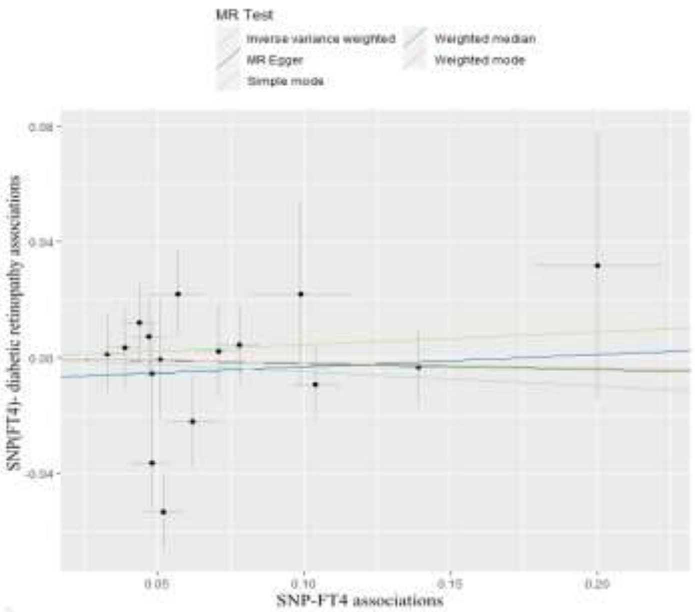

A. Scatter plot of causal effect of FT4 on DR

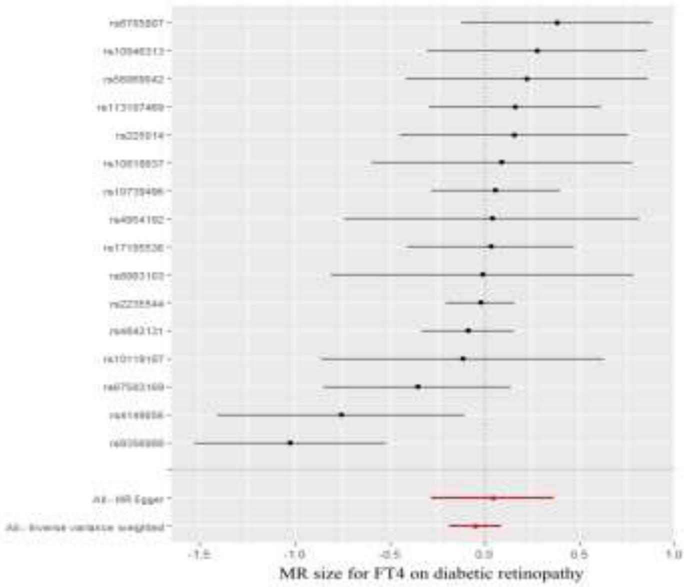

B. Forest plot of causal effect of FT4 on DR

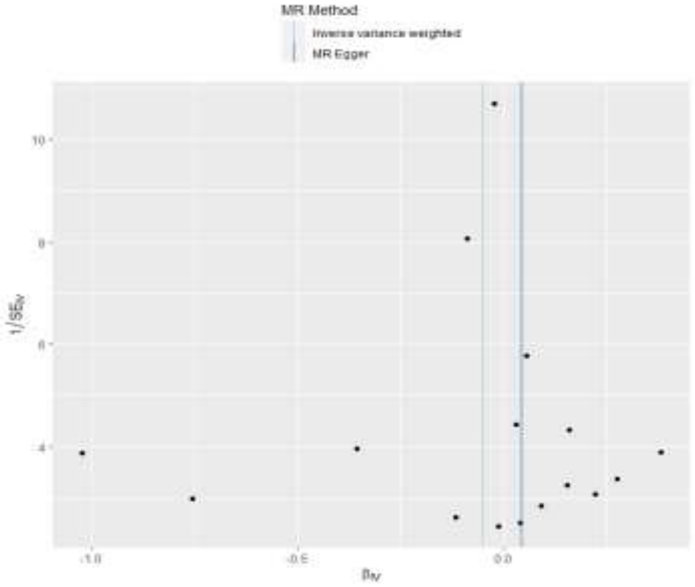

C. Funnel plot of causal effect of FT4 on DR

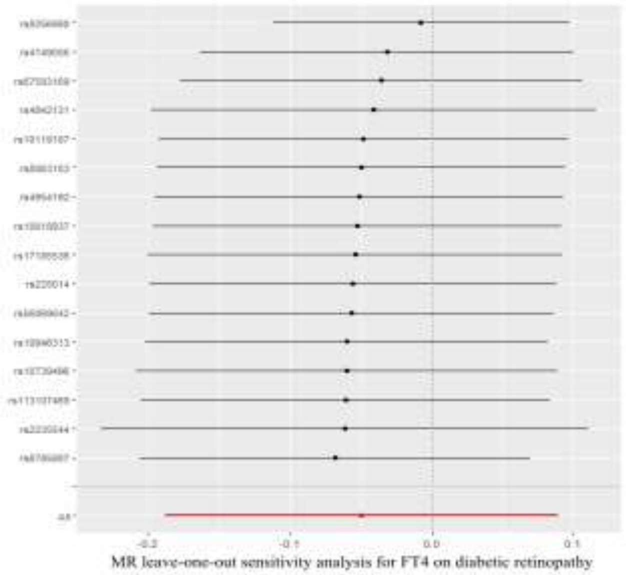

D. Leave-one-out analysis for IVW-RE MR of FT4 on DR

eFigure 5. TSH-associated SNPs with risk of DR

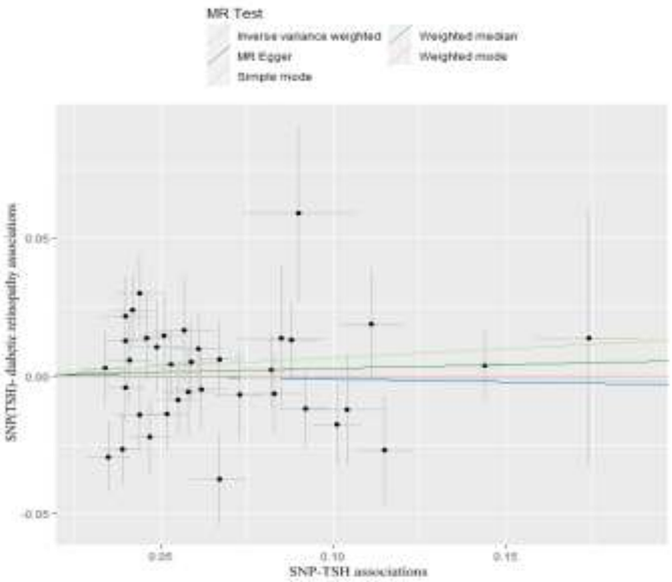

A. Scatter plot of causal effect of TSH on DR

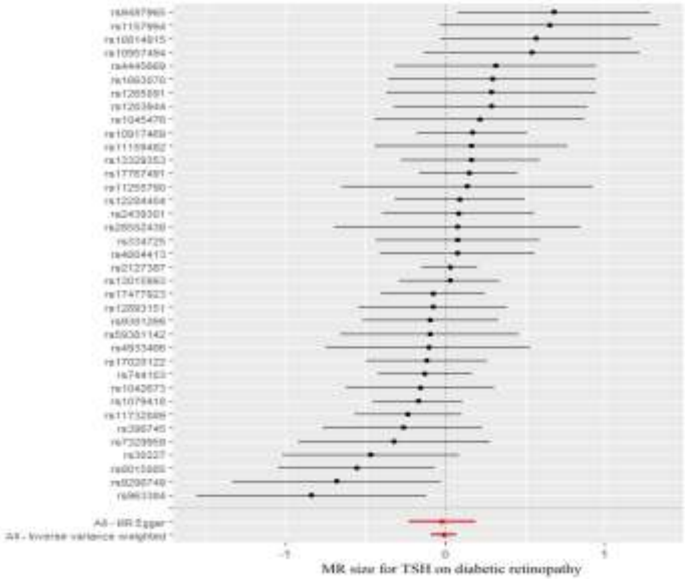

B. Forest plot of causal effect of TSH on DR

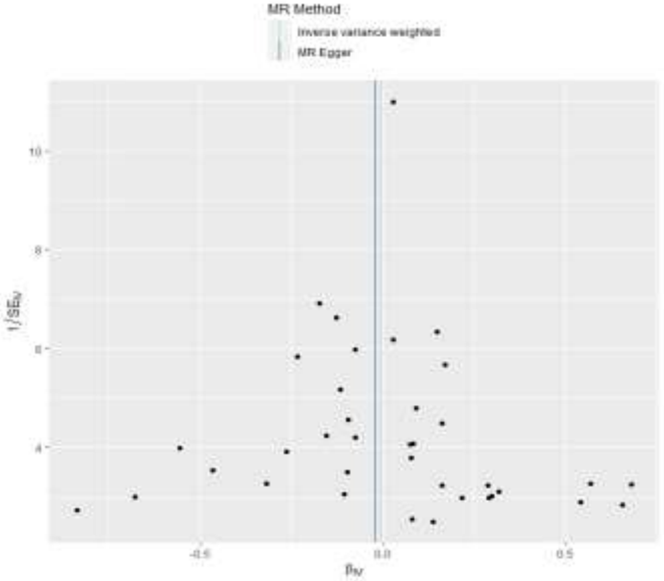

C. Funnel plot of causal effect of TSH on DR

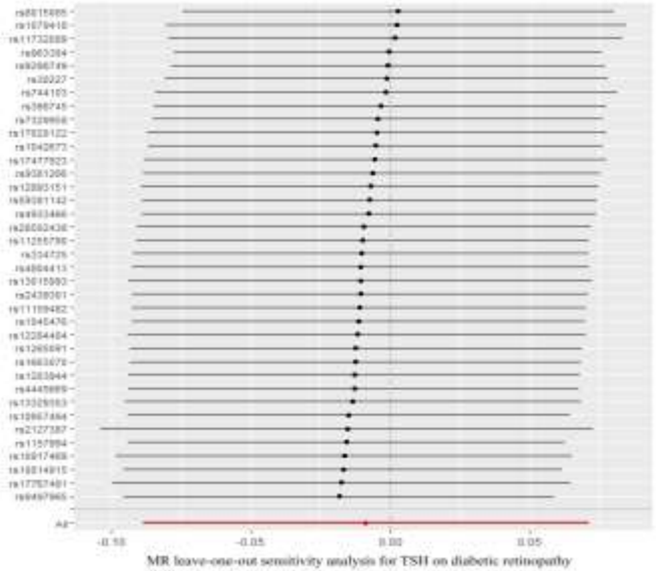

D. Leave-one-out analysis for IVW-RE MR of TSH on DR

eFigure 6. TPOAb-associated SNPs with risk of DR

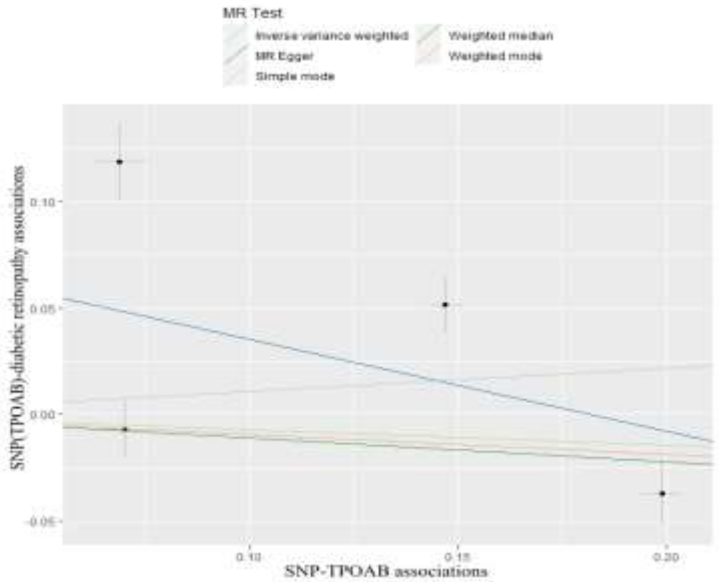

A. Scatter plot of causal effect of TPOAb on DR

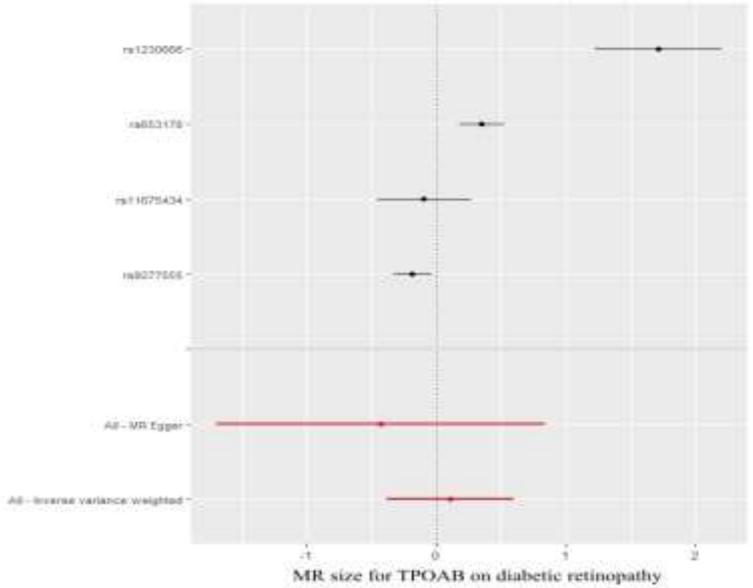

B. Forest plot of causal effect of TPOAb on DR

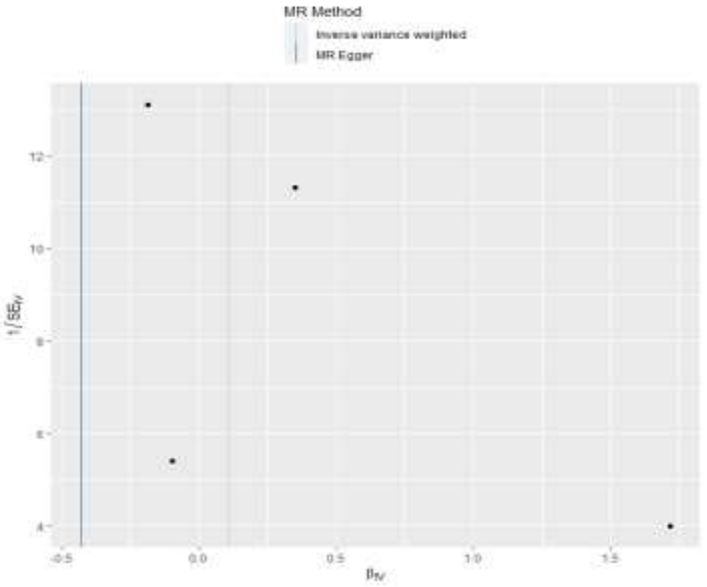

C. Funnel plot of causal effect of TPOAb on DR

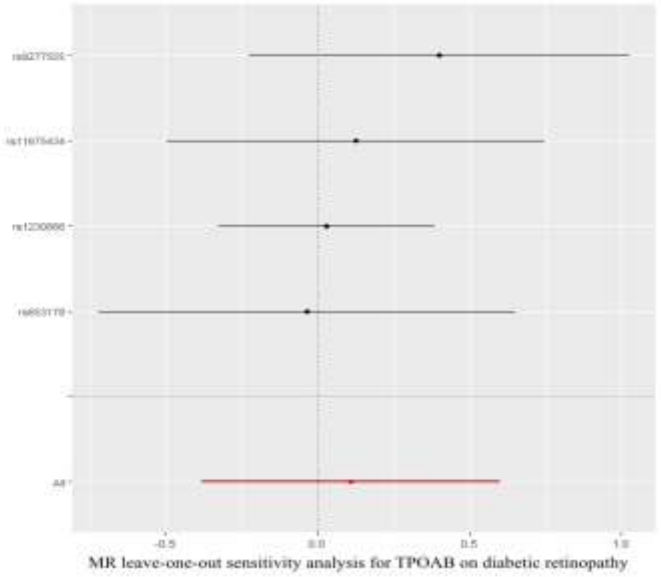

D. Leave-one-out analysis for IVW-RE MR of TPOAb on DR

eFigure 7. FT4-associated SNPs with risk of eGFR

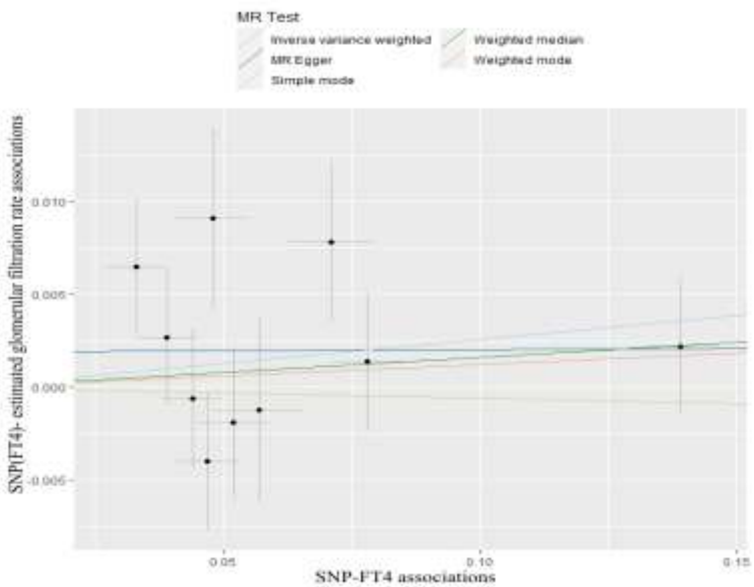

A. Scatter plot of causal effect of FT4 on eGFR

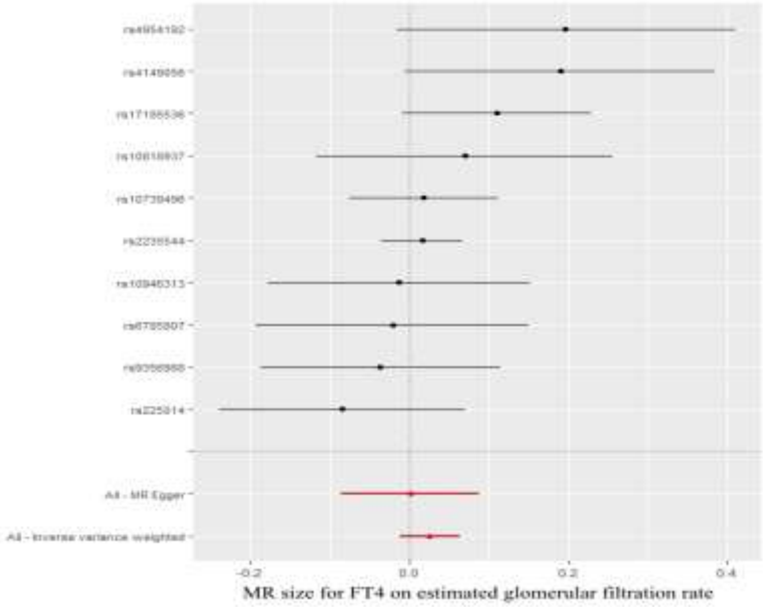

B. Forest plot of causal effect of FT4 on eGFR

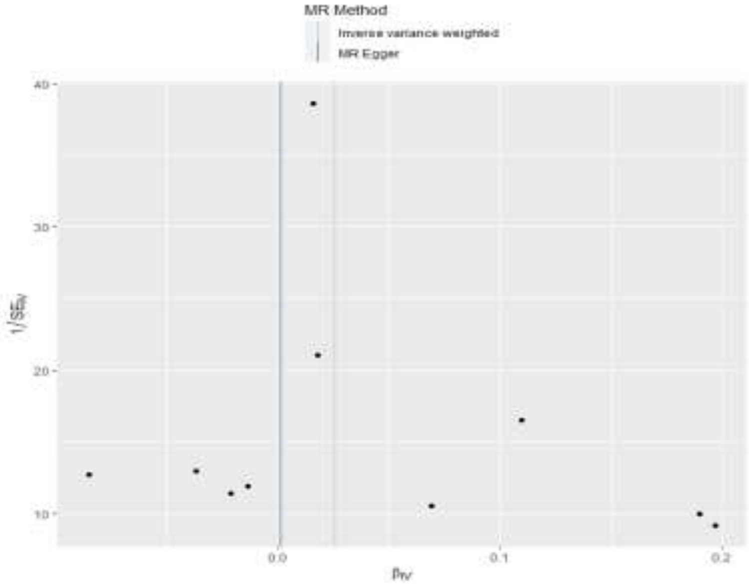

C. Funnel plot of causal effect of FT4 on eGFR

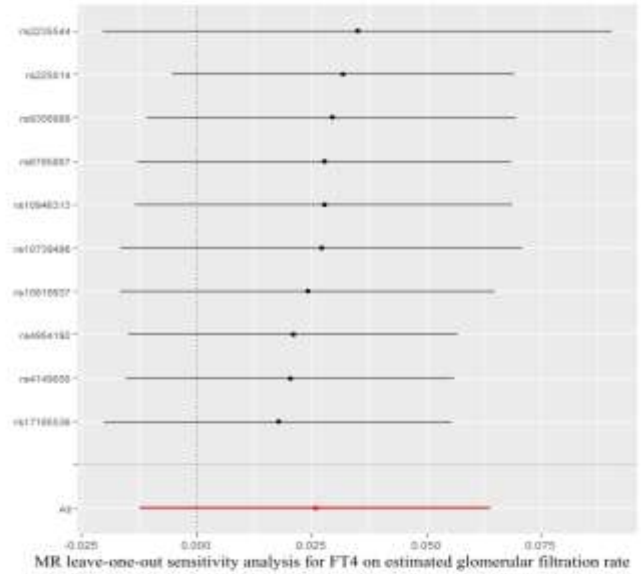

D. Leave-one-out analysis for IVW-RE MR of FT4 on eGFR

eFigure 8. TSH-associated SNPs with risk of eGFR

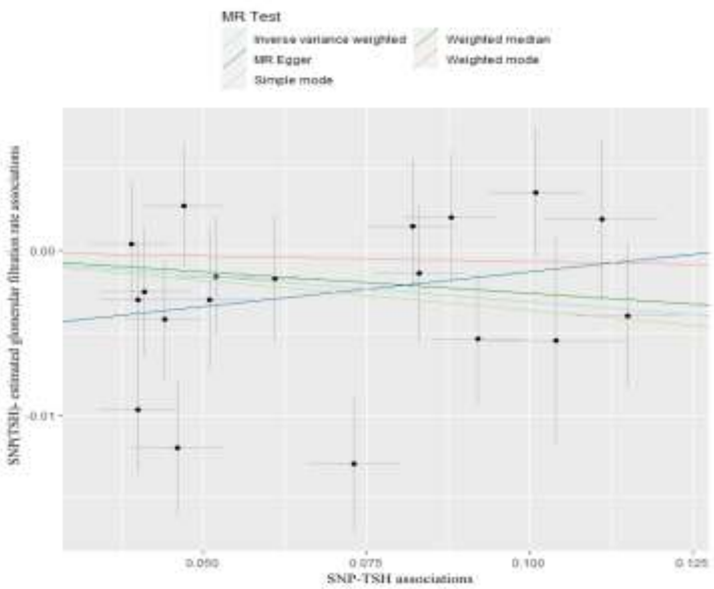

A. Scatter plot of causal effect of TSH on eGFR

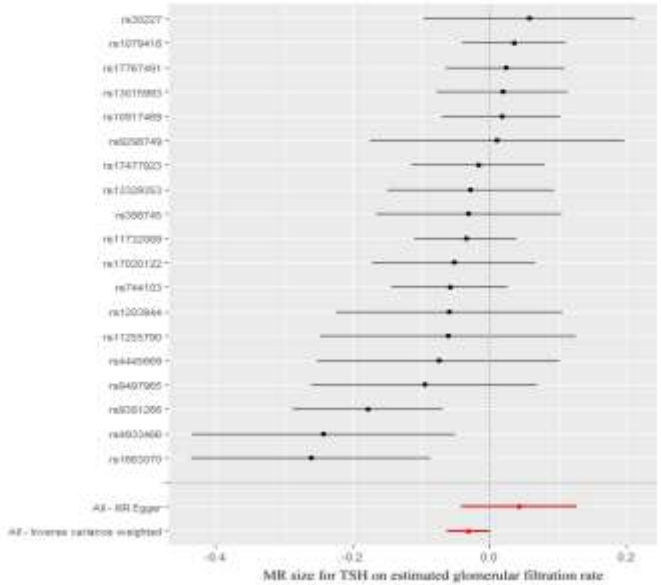

B. Forest plot of causal effect of TSH on eGFR

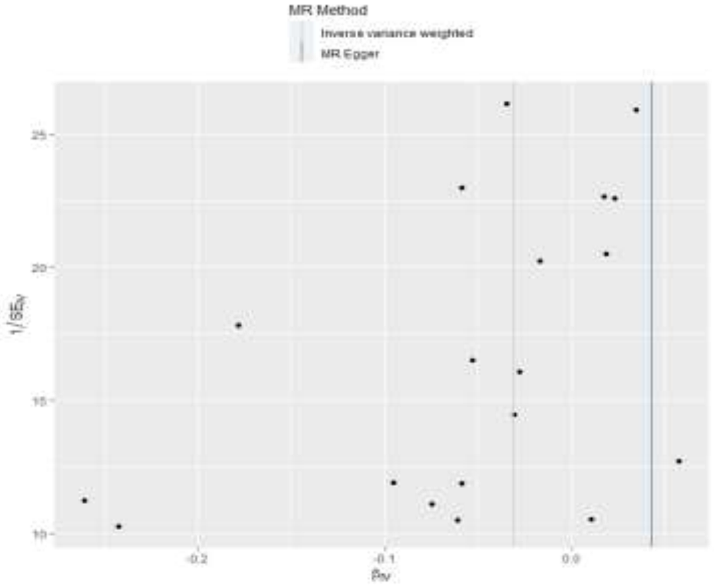

C. Funnel plot of causal effect of TSH on eGFR

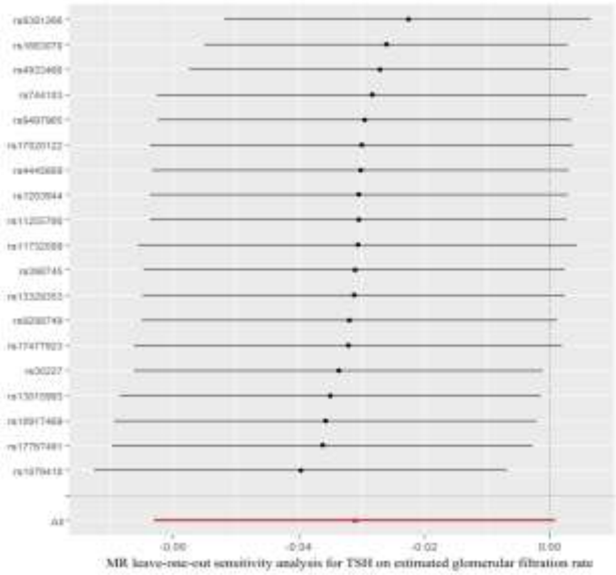

D. Leave-one-out analysis for IVW-RE MR of TSH on eGFR

eFigure 9. TPOAb-associated SNPs with risk of eGFR

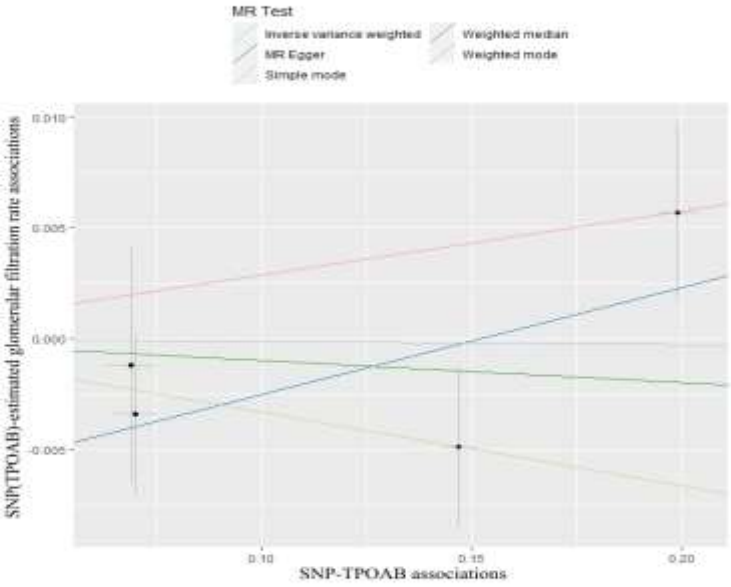

A. Scatter plot of causal effect of TPOAb on eGFR

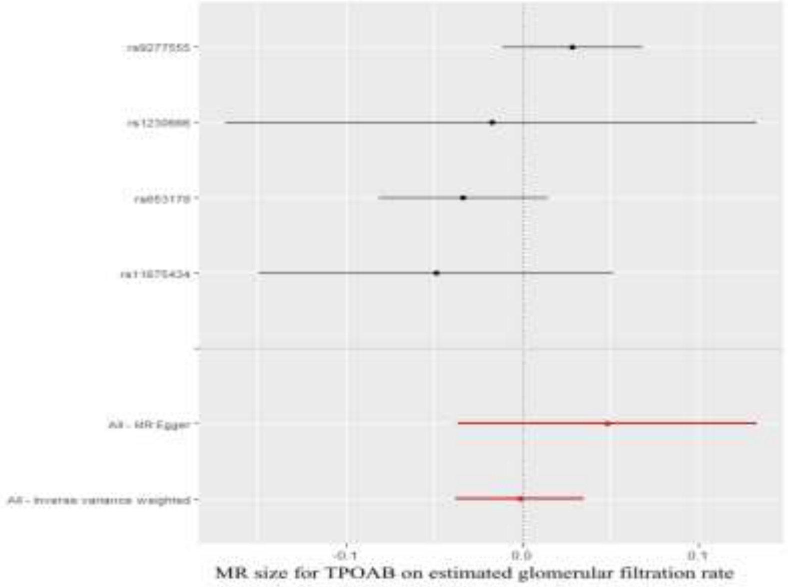

B. Forest plot of causal effect of TPOAb on eGFR

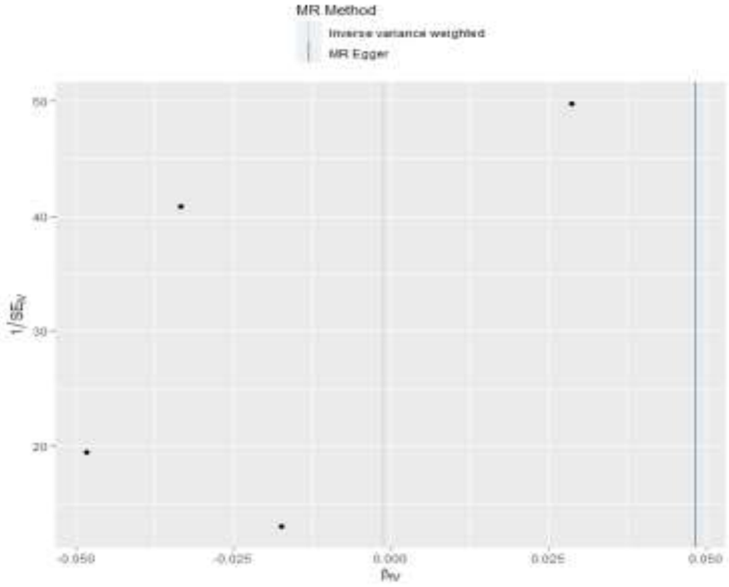

C. Funnel plot of causal effect of TPOAb on eGFR

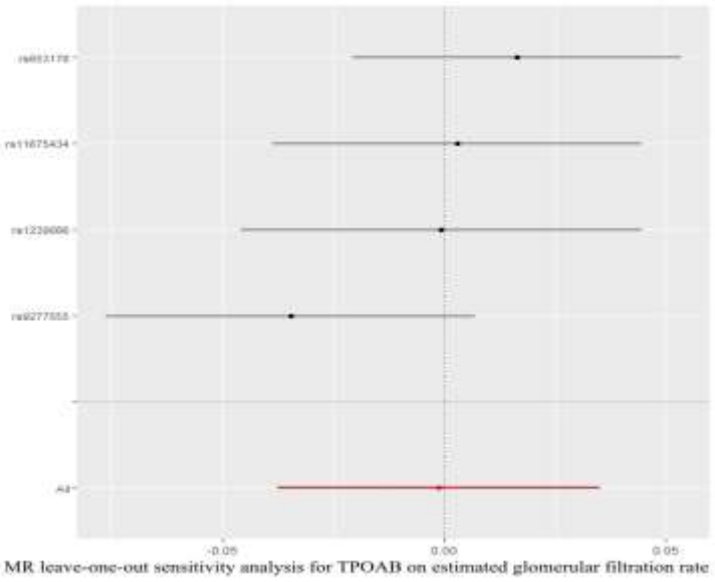

D. Leave-one-out analysis for IVW-RE MR of TPOAb on eGFR

eFigure 10. FT4-associated SNPs with risk of ACR

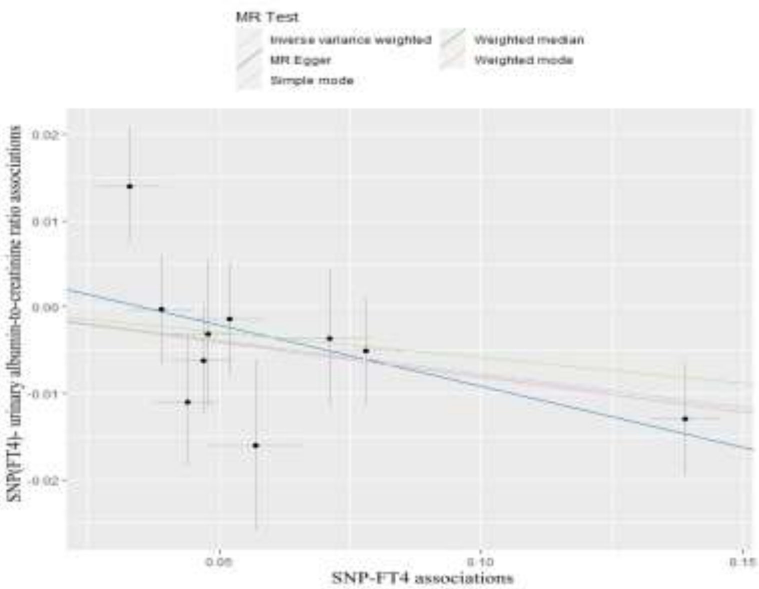

A. Scatter plot of causal effect of FT4 on ACR

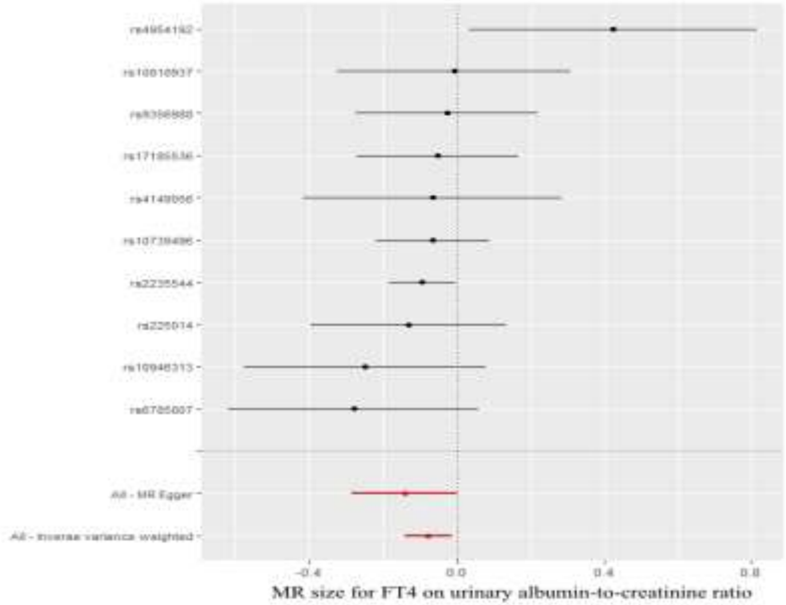

B. Forest plot of causal effect of FT4 on ACR

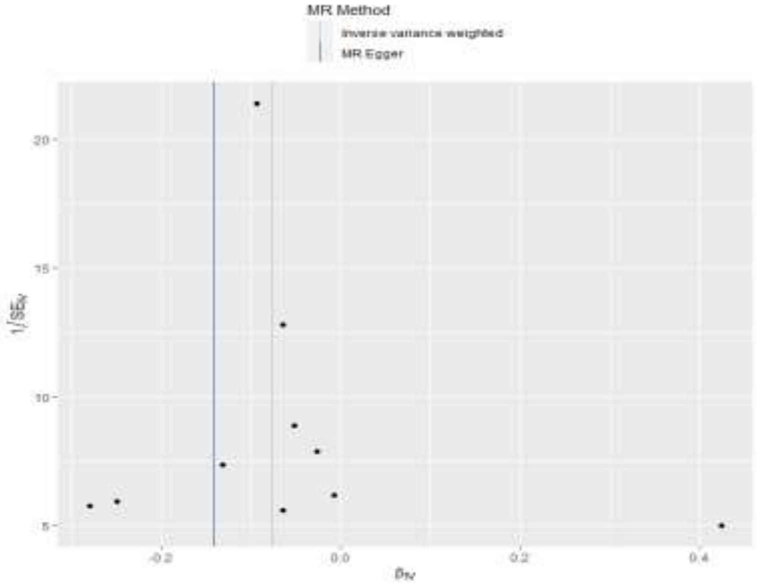

C. Funnel plot of causal effect of FT4 on ACR

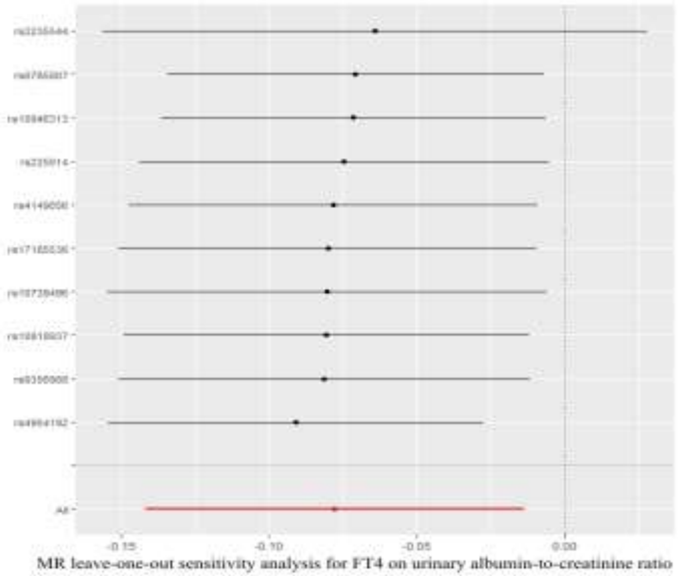

D. Leave-one-out analysis for IVW-RE MR of FT4 on ACR

eFigure 11.TSH-associated SNPs with risk of ACR

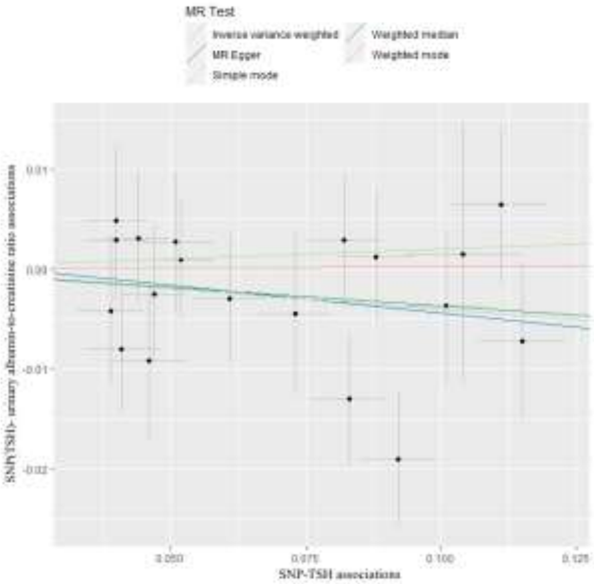

A. Scatter plot of causal effect of TSH on ACR

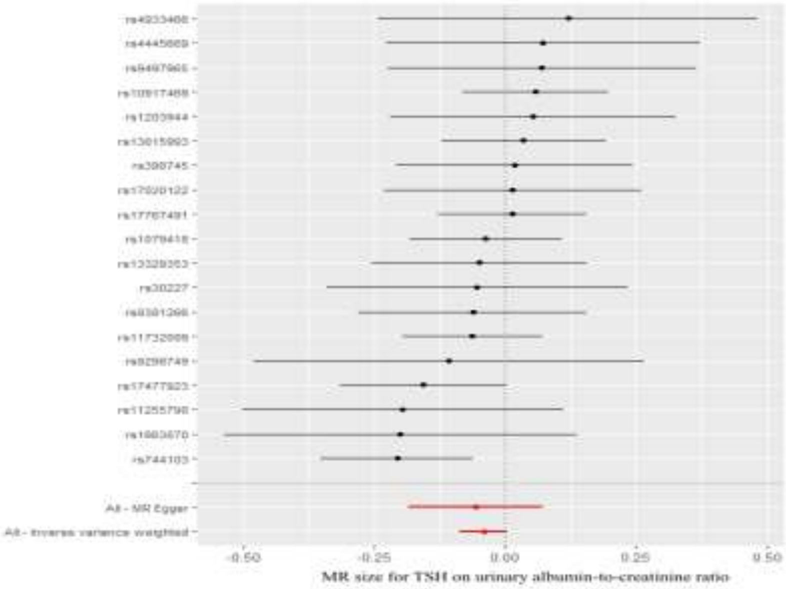

B. Forest plot of causal effect of TSH on ACR

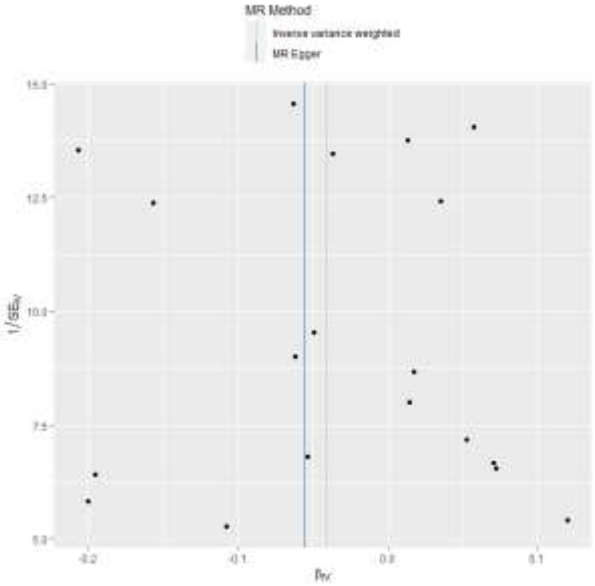

C. Funnel plot of causal effect of TSH on ACR

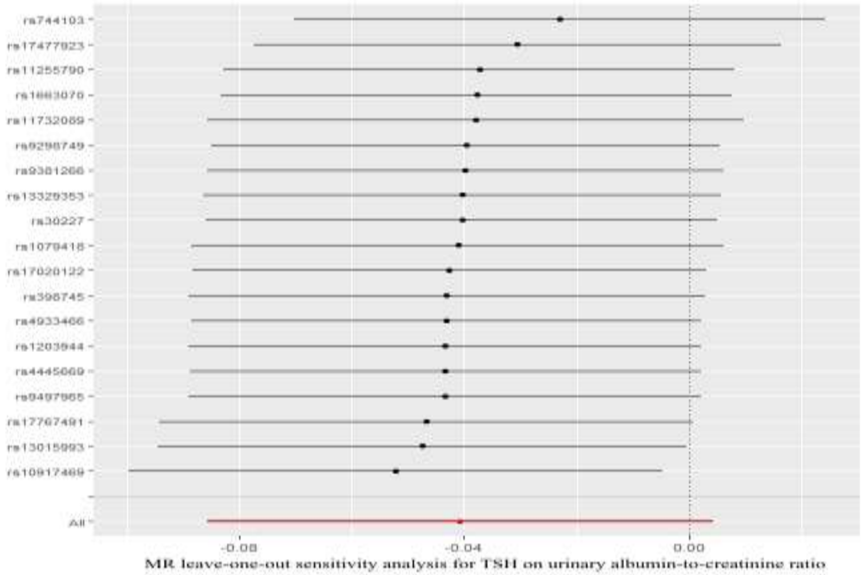

D. Leave-one-out analysis for IVW-RE MR of TSH on ACR

eFigure 12.TPOAb-associated SNPs with risk of ACR

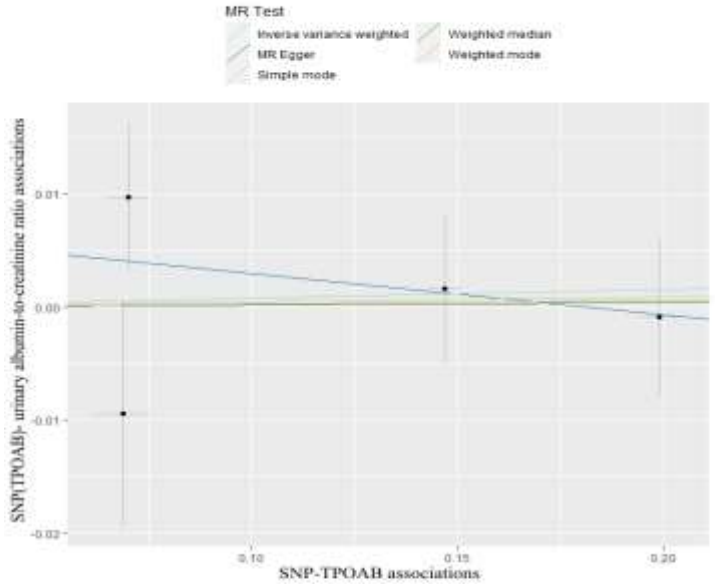

A. Scatter plot of causal effect of TPOAb on ACR

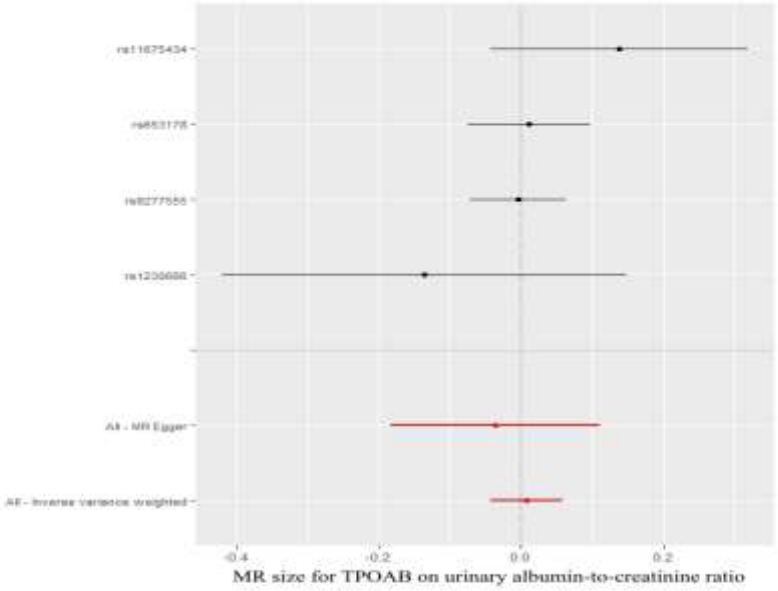

B. Forest plot of causal effect of TPOAb on ACR

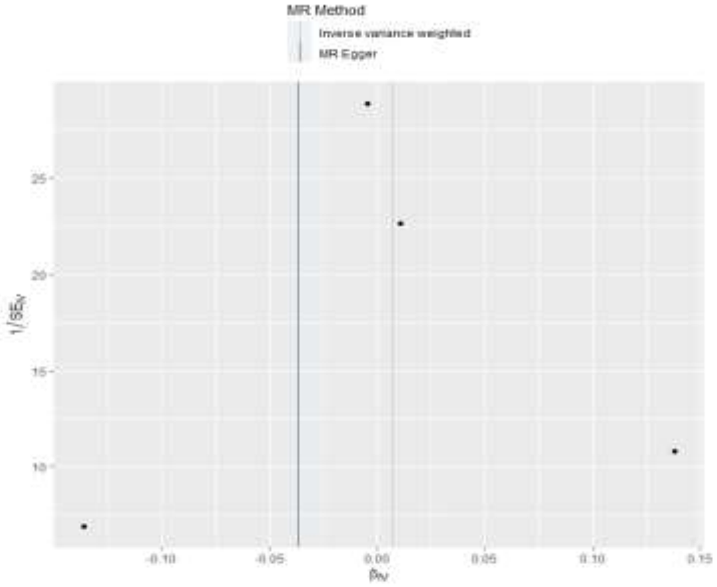

C. Funnel plot of causal effect of TPOAb on ACR

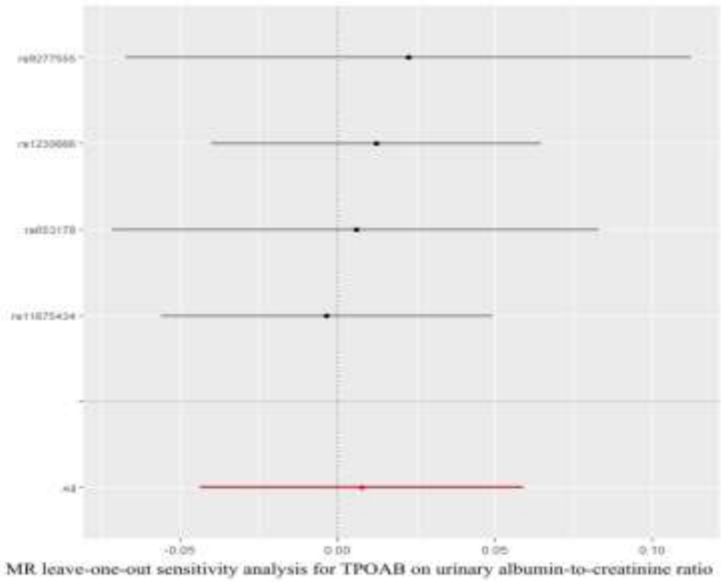

D. Leave-one-out analysis for IVW-RE MR of TPOAb on ACR

eFigure 13. FT4-associated SNPs with risk of PDR

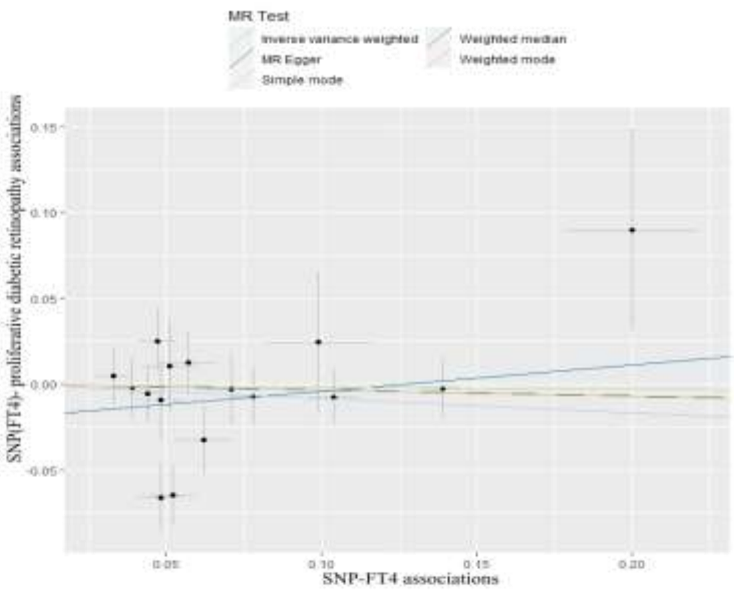

A. Scatter plot of causal effect of FT4 on PDR

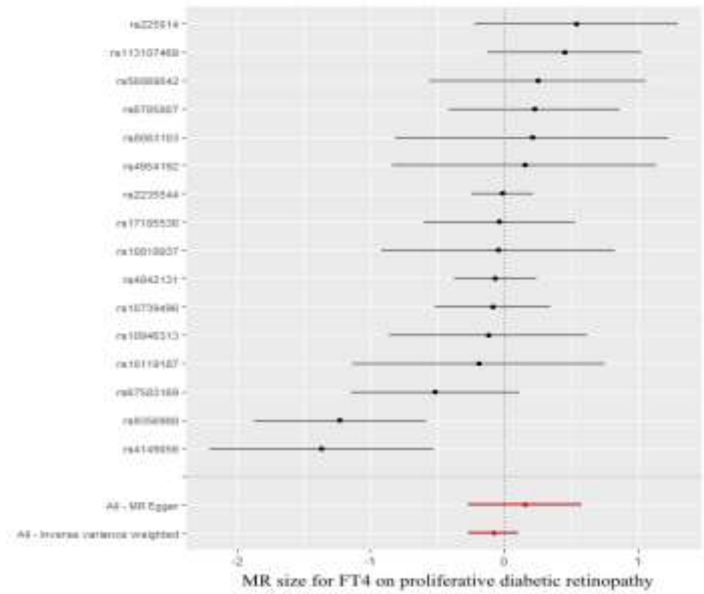

B. Forest plot of causal effect of FT4 on PDR

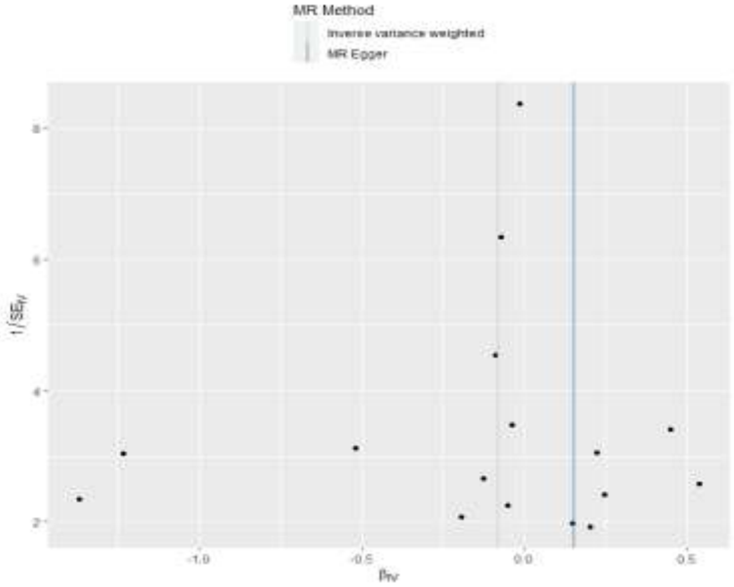

C. Funnel plot of causal effect of FT4 on PDR

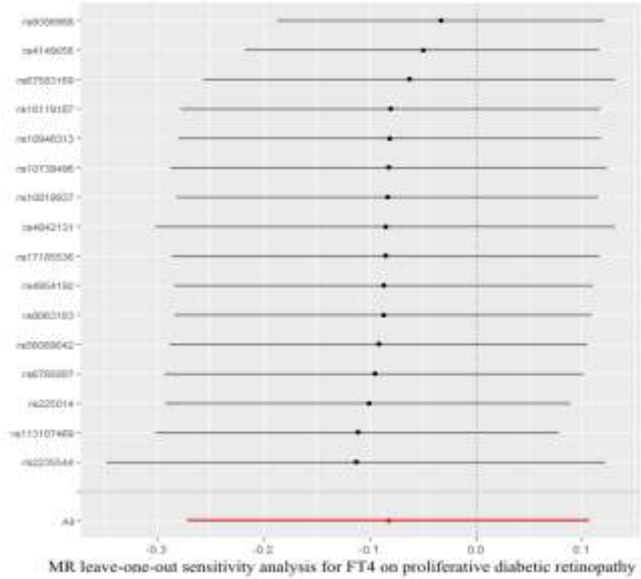

D. Leave-one-out analysis for IVW-RE MR of FT4 on PDR

eFigure 14. TSH-associated SNPs with risk of PDR

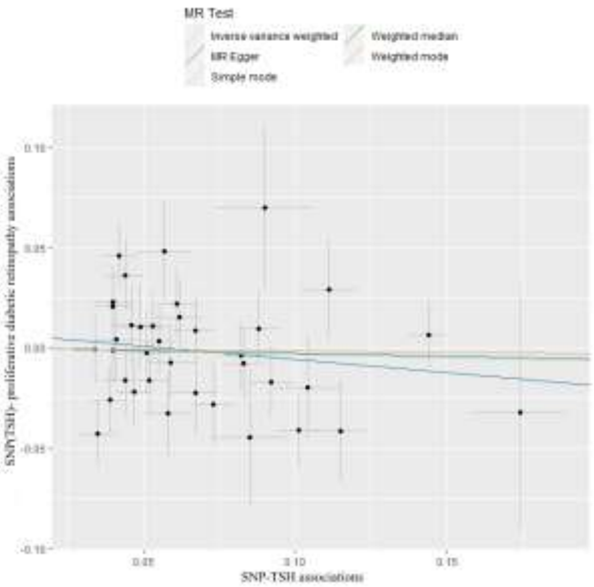

A. Scatter plot of causal effect of TSH on PDR

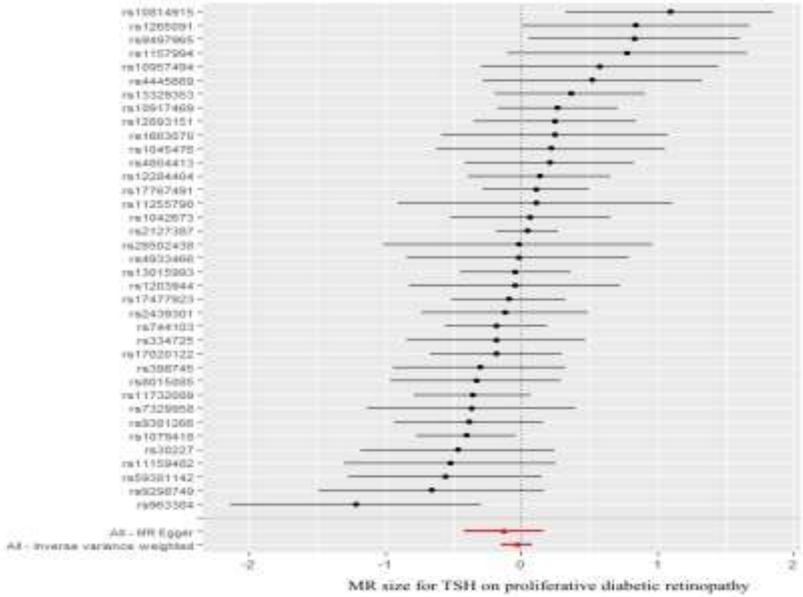

B. Forest plot of causal effect of TSH on PDR

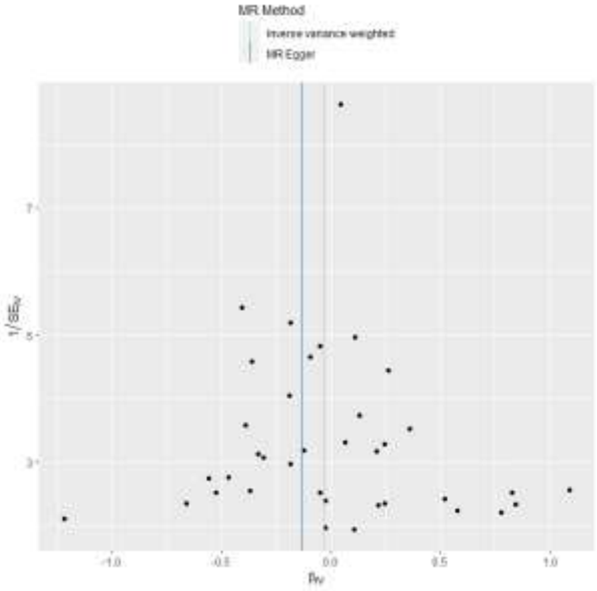

C. Funnel plot of causal effect of TSH on PDR

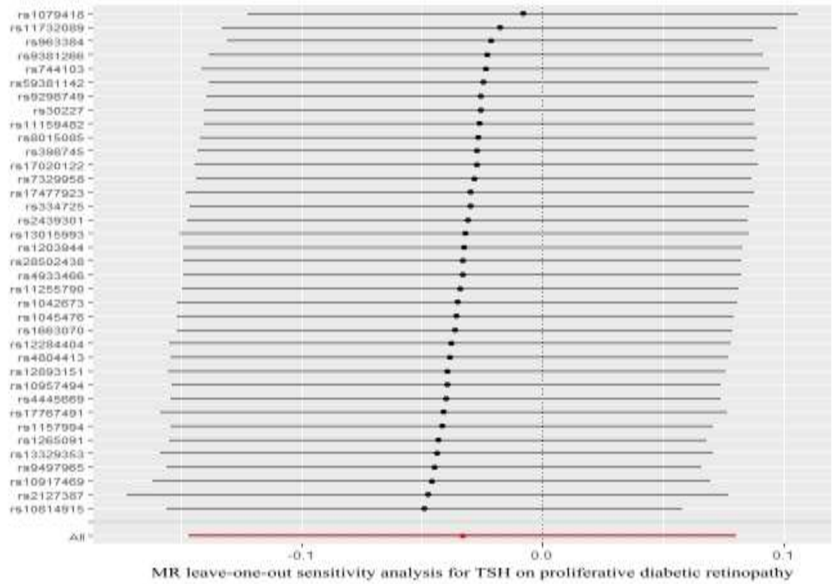

D. Leave-one-out analysis for IVW-RE MR of TSH on PDR

eFigure 15. TPOAb-associated SNPs with risk of PDR

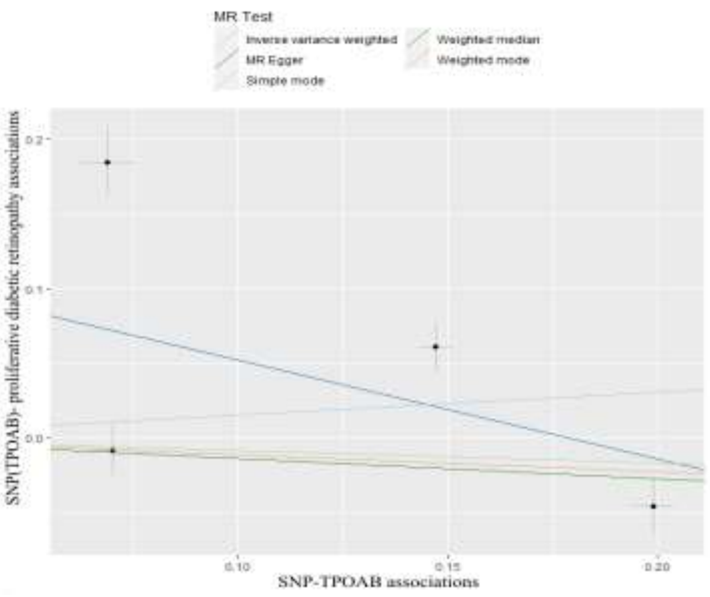

A. Scatter plot of causal effect of TPOAb on PDR

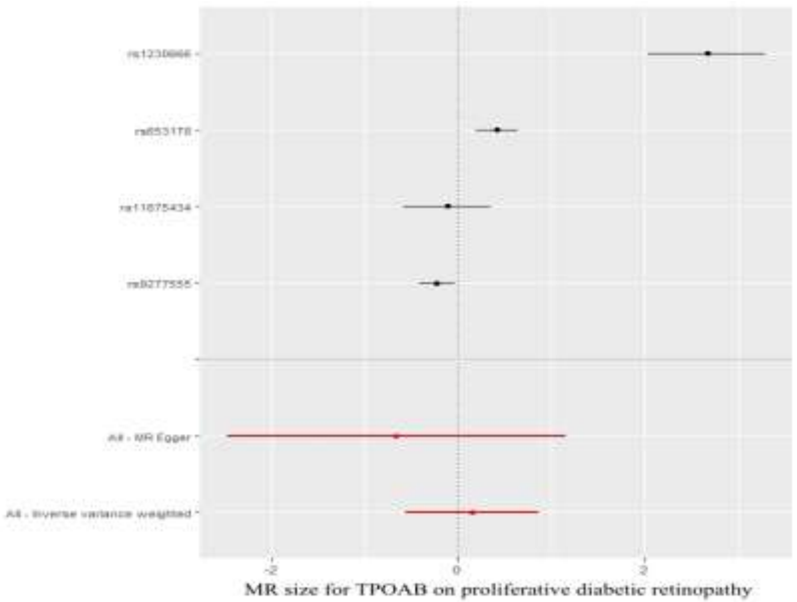

B. Forest plot of causal effect of TPOAb on PDR

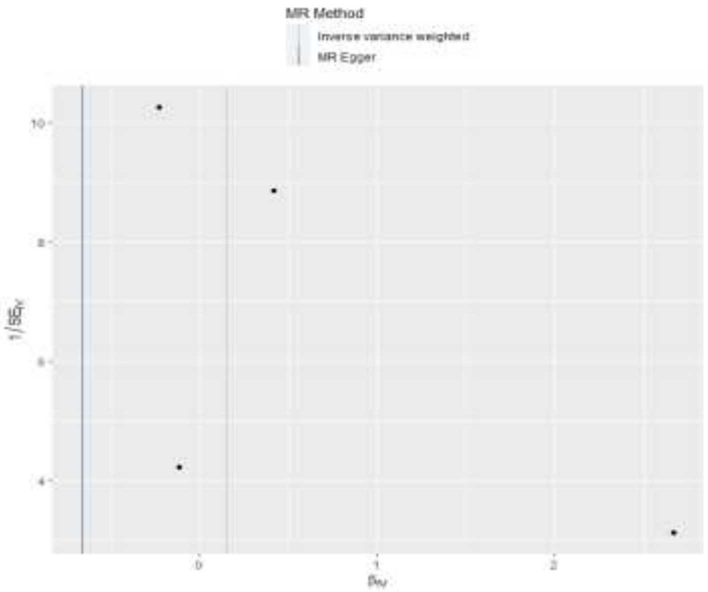

C. Funnel plot of causal effect of TPOAb on PDR

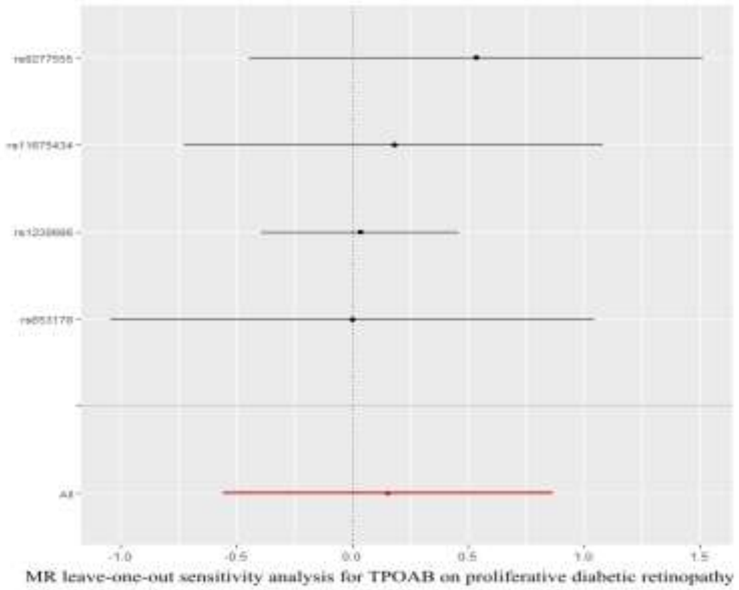

D. Leave-one-out analysis for IVW-RE MR of TPOAb on PDR

Reference list

1. Teumer A, Chaker L, Groeneweg S, et al. Genome-wide analyses identify a role for SLC17A4 and AADAT in thyroid hormone regulation. Nat Commun. 2018;9(1):4455.
2. Panicker V, Cluett C, Shields B, et al. A common variation in deiodinase 1 gene DIO1 is associated with the relative levels of free thyroxine and triiodothyronine. J Clin Endocrinol Metab. 2008;93(8):3075-3081.
3. Schultheiss UT, Teumer A, Medici M, et al. A genetic risk score for thyroid peroxidase antibodies associates with clinical thyroid disease in community-based populations. J Clin Endocrinol Metab. 2015;100(5):E799-E807.
4. Medici M, Porcu E, Pistis G, et al. Identification of novel genetic loci associated with thyroid peroxidase antibodies and clinical thyroid disease. PLoS Genet. 2014;10(2):e1004123.
